# *Deletion of Gpr27 in vivo* reduces insulin mRNA but does not result in diabetes

**DOI:** 10.1038/s41598-020-62358-4

**Published:** 2020-03-27

**Authors:** Deeksha G. Chopra, Nicholas Yiv, Thomas G. Hennings, Yaohuan Zhang, Gregory M. Ku

**Affiliations:** 1 Diabetes Center, UCSF, San Francisco, CA 94143 USA; 2 Biomedical Sciences Graduate Program, UCSF, San Francisco, CA 94143 USA; 3 Metabolic Biology Graduate Program, UCB, Berkeley, CA 94720 USA; 4 Division of Endocrinology and Metabolism, Department of Medicine, UCSF, San Francisco, CA 94143 USA

**Keywords:** Gene expression, Type 2 diabetes

## Abstract

*Gpr27* is a highly conserved, orphan G protein coupled receptor (GPCR) previously implicated in pancreatic beta cell insulin transcription and glucose-stimulated insulin secretion *in vitro*. Here, we characterize a whole-body mouse knockout of *Gpr27*. *Gpr27* knockout mice were born at expected Mendelian ratios and exhibited no gross abnormalities. Insulin and *Pdx1* mRNA in *Gpr27* knockout islets were reduced by 30%, but this did not translate to a reduction in islet insulin content or beta cell mass. *Gpr27* knockout mice exhibited slightly worsened glucose tolerance with lower plasma insulin levels while maintaining similar insulin tolerance. Unexpectedly, *Gpr27* deletion reduced expression of *Eif4e3*, a neighboring gene, likely by deleting transcription start sites on the anti-sense strand of the *Gpr27* coding exon. Our data confirm that loss of *Gpr27* reduces insulin mRNA *in vivo* but has only minor effects on glucose tolerance.

## Introduction

Pancreatic beta-cells play a central role in regulating blood glucose homeostasis by secreting precise amounts of insulin in response to blood glucose concentrations. Beta-cell death or dysfunction contributes to the pathogenesis of both type 1 and type 2 diabetes mellitus. Since current diabetes therapies are often insufficient to manage hyperglycemia, novel strategies are needed. Therapies to improve beta cell function and survival are an attractive approach to generate new therapies for diabetes.

GPCRs have a proven track record of being viable drug targets. Thirty-four percent of currently approved therapies target 103 different GPCRs^1^. In diabetes, the blockbuster GLP1R agonists^2^ and the dipeptidyl peptidase IV inhibitors^3^ are prominent examples of successful drugs targeting a beta cell GPCR. Nonetheless, many GPCRs remain orphan receptors and are poorly characterized; these could be future diabetes drug targets^4^.

We previously identified the highly conserved GPCR *Gpr27* (also known as SREB1, or super conserved receptor in brain 1) as a positive regulator of both insulin transcription and insulin secretion during a loss of function screen conducted in the MIN6 pancreatic beta cell line^5^. *Gpr27* is predominantly expressed in the brain, pituitary, and the pancreatic beta cell^5^ (GTEx Portal). While this previous work indicates that *Gpr27* may represent a new therapeutic target for diabetes, *Gpr27* remains uncharacterized *in vivo*.

To explore the role of *Gpr27* in glucose homeostasis *in vivo*, we studied a whole body *Gpr27* knockout mouse. Islets from *Gpr27* knockout mice had lower mRNA expression of insulin mRNA but unchanged insulin protein. *Gpr27* knockout mice had reduced body mass, had modest worsening of glucose intolerance, and had decreased plasma insulin levels. This work adds to our understanding of the role of *Gpr27* in regulating glucose homeostasis *in vivo*.

## Results

**Figure 1:**
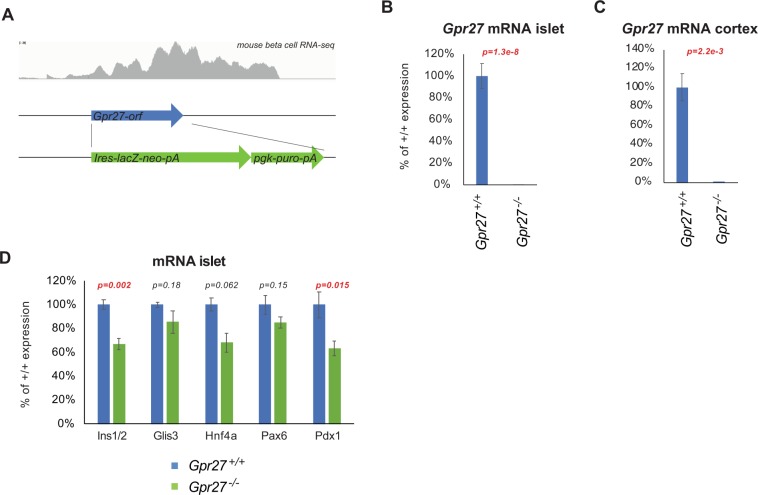
A global *Gpr27* knockout mouse has reduced islet insulin and *Pdx1* mRNA. (**A**) The *Gpr27* locus contains a single exon (blue). The top panel shows polyA RNA-seq reads from sorted primary mouse beta cells^16^ The targeting vector (bottom) replaces the single coding exon of *Gpr27* with an internal ribosomal entry site, beta-galactosidase (lacZ)-neomycin (neo) resistance cassette and polyadenylation signal (pA). This is followed by a 3-phosphoglycerate kinase promoter puromycin resistance cassette (pgk-puro-pA). (**B**) *Gpr27* mRNA measured by RT-qPCR from isolated islets of 9 to 17-week old *Gpr27* knockout animals or their wild type littermates. n = 9 wt, 11 ko. (**C**) *Gpr27* mRNA measured by RT-qPCR from cerebral cortex, n = 3 wt, 3 ko. (**D**) As in 1B but for *Ins1/2, Glis3, Hnf4a, Pax6* and *Pdx1* mRNA. For *Ins1/2*, *Pax6*, and *Hnf4a*, n = 6 wt, 8 ko. For *Glis3* n = 9 wt, 10 ko. For *Pdx1*, n = 10 wt, 11 ko. Error bars indicate SEM. p values were calculated by Student’s t-test and corrected for multiple testing by the Benjamini-Hochberg method (part D).

*Gpr27* knockout mice were generated by the Texas A&M Institute for Genomic Medicine (TIGM) by replacing the *Gpr27* coding exon with an a beta galactosidase-neomycin fusion protein and a 3-phosphoglyerate kinase promoter driving puromycin resistance cassette in embryonic stem cells (Fig. 1A and Methods). Crosses of heterozygous *Gpr27* mice revealed all 3 genotypes at the expected Mendelian ratios (89 wild type, 154 heterozygous, 77 knockouts at 3 weeks of age, chi-squared p value = 0.509) and displayed no gross abnormalities.

*Gpr27* mRNA was undetectable in islets isolated from *Gpr27* knockout mice (Fig. 1B). *Gpr27* mRNA was also undetectable in the cerebral cortex of *Gpr27* knockout mice (Fig. 1C). As we initially identified *Gpr27* as a positive regulator of insulin transcription, we measured mature insulin mRNA by quantitative PCR (Fig. 1D). Confirming our *in vitro* data^5^, we found that insulin mRNA was reduced by ~30% in *Gpr27* knockout islets. Levels of *Pdx1* were also reduced by ~30% in *Gpr27* knockout mice while *Glis3*, *Pax6*, and *Hnf4a* were not changed (Fig. 1D).

**Figure 2:**
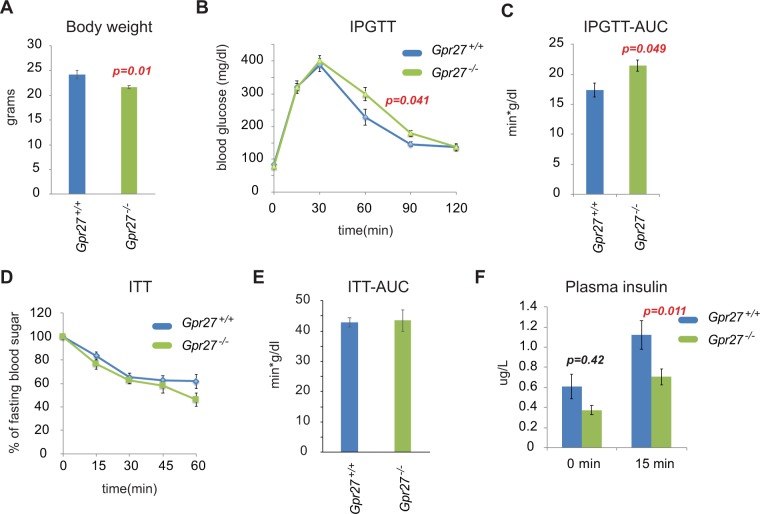
*Gpr27* knockout mice have modestly impaired glucose tolerance and plasma insulin levels but normal insulin sensitivity on a chow diet. (**A**) Body weight at 12 weeks of age. (**B**) Intraperitoneal glucose tolerance test at 12 weeks of age. For time, F(2.612,211.599), p < 0.001; for time*genotype F(2.612, 3.167), p = 0.041. (**C**) Area under the curve for B. (**D**) Insulin tolerance test at 12 weeks of age. For time, F(2.139,53.5666), p < 0.001; for time*genotype F(2.139, 0.402), p = 0.686. (**E**) Area under the curve for D. **(F)** Plasma insulin at 12 weeks of age. For time, F(1,122.958), p < 0.001. For time*genotype, F(1, 5.730), p = 0.03. For B, E and D, Mauchly’s test of sphericity was violated so these were Greenhouse-Geisser corrected. Error bars show SEM. *p < 0.05. n = 8 wt, 9 ko.

**Figure 3:**
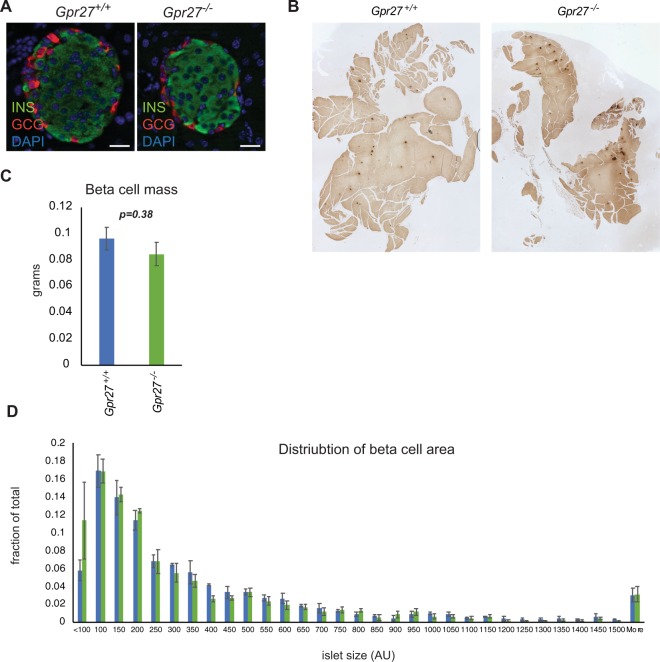
*Gpr27* knockout mice have normal beta cell mass and islet size. (**A**) Normal islet morphology in *Gpr27* knockout islets. Sections were stained for insulin and glucagon and DNA. Representative of 2 mice per genotype. (**B**) Representative pancreas sections from wild type and *Gpr27* knockout animals stained for insulin (dark brown). (**C**) Beta cell mass at 20–23 weeks of age, n = 4 wt, 4 ko. (**D**) Histogram of islet size, n = 4 wt, 4, ko. Error bars show SEM. p-value by Student’s t-test.

At 12 weeks of age, *Gpr27* mice were 10% lighter than their wild type littermates (Fig. 2A). Despite their lower average weight, we found that *Gpr27* knockout animals had slightly impaired glucose tolerance compared to their wild type littermates (two-way mixed ANOVA, time*genotype p = 0.048) that just passed our significance threshold of 0.05 (Fig. 2B). Area under the curve (AUC) analysis of blood glucose measurements confirmed this impaired glucose tolerance in *Gpr27* knockout mice (p = 0.049) (Fig. 2C). Insulin tolerance testing was not significantly different between wild type and knockout animals (Fig. 2D,E). Based on our prior studies of *Gpr27* in beta cell lines, we predicted *Gpr27* knockout mice might have reduced insulin secretion. Indeed, plasma insulin levels were reduced in the knockout animals (two-way mixed ANOVA, time*genotype p = 0.03) (Fig. 2F), and this was due to a significant reduction in the plasma insulin measured 15 minutes after glucose challenge (p = 0.011). Islet morphology from *Gpr27* knockout mice was largely normal as determined by immunofluorescence staining for insulin and glucagon (Fig. 3A). Beta cell mass and the distribution of islet sizes were unchanged. (Fig. 3B–D).

**Figure 4:**
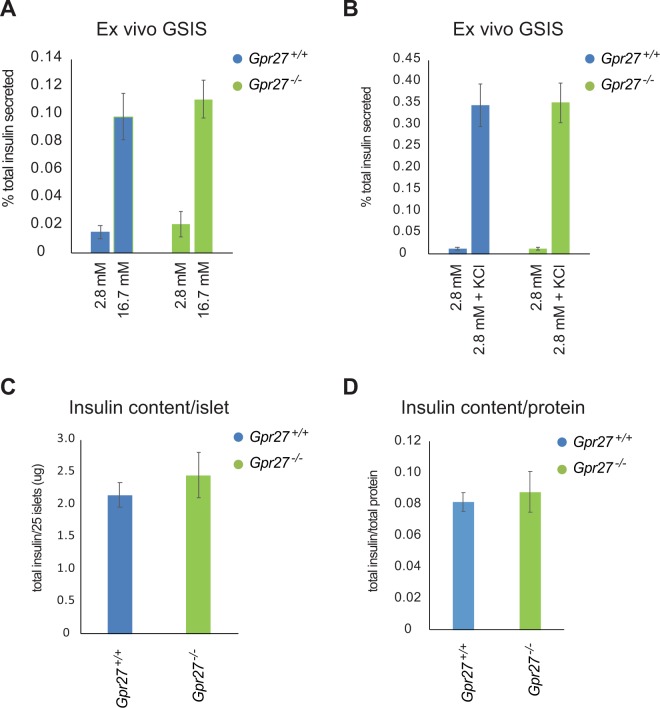
*Ex vivo* GSIS of *Gpr27* knockout islets is normal. (**A**) *Ex vivo* GSIS of male 9 to 17-week old *Gpr27* wild type and *Gpr27* knockout islets in response to 2.8 mM and 16.7 mM glucose. n = 5 wt, 6 ko mice. (**B**) *Ex vivo* GSIS of male 9 to 17-week old *Gpr27* wild type and *Gpr27* knockout islets in response to 2.8 mM and 2.8 mM glucose and 40 mM KCl. n = 5 wt, 6 ko mice. (**C**) Total islet insulin from 25 islets of each genotype. n = 5 wt, 6 ko. (**D**) As in 4 C except normalized to total protein content. Error bars indicate standard error.

Islets isolated from *Gpr27* mice did not display a detectable decrease in fractional insulin secretion in response to glucose (Fig. 4A) or potassium chloride stimulation (Fig. 4B). Despite the fact that insulin mRNA was reduced, there was also no detectable change in amount of insulin protein when normalized to islet number or to total protein concentration (Fig. 4C,D).

**Figure 5:**
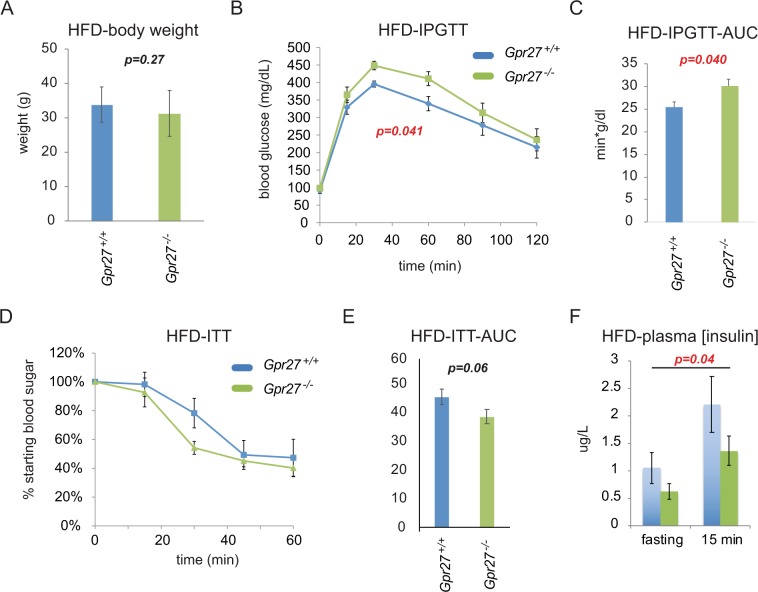
*Gpr27* knockout mice are modestly hyperglycemic on a high fat diet with unchanged insulin sensitivity. (**A**) Body weight at 12 weeks after HFD. (**B**) IPGTT. For time, F(2.612, 211.599) p < 0.001. time*genotype F(2.612,3.167) p = 0.041. n = 9 wt mice, 12 ko mice. (**C**) Area under the curve for IPGTT. (**D**) Insulin tolerance test. n = 7 wt mice, 9 ko mice. For time, F(2.366,77.525), p < 0.001. For time*genotype F(2.366, 1.985), p = 0.147. (**E**) AUC for insulin tolerance test. SEM is shown. (**F**) Plasma insulin concentrations as in 2 F; Two way ANOVA: time, F(1, 9.252), p = 0.006, for genotype F(1,4.371), p = 0.04. For time*genotype F(1,0.465), p = 0.5. n = 7 ko and 4 wt. For B and F, Mauchly’s test of sphericity was violated so these were Greenhouse-Geisser corrected. Error bars show SEM.

To confirm impaired glucose tolerance, we challenged the mice with increased insulin resistance by placing an independent cohort of *Gpr27* knockout mice and wild type littermate controls on a high fat diet from 3 weeks to 12 weeks of age. After high fat feeding, the body weights of *Gpr27* and wild type mice were not different (Fig. 5A). The glucose tolerance and the glucose AUC were again statistically significantly changed in the *Gpr27* knockout mice (Fig. 5B,C). Insulin sensitivity exhibited a trend towards being improved in the knockout animals that did not reach our statistical significance threshold, further implying an underlying an insulin secretory defect (Fig. 5D,E). Plasma insulin levels were significantly lower in the *Gpr27* knockout mice (2-way ANOVA for genotype p = 0.04) (Fig. 5F). This effect came from both the fasting and 15-minute time points as the p value for interaction (time*genotype) was not significant and the post-hoc test between genotypes at 0 and 15 minutes did not reach the significance threshold (p = 0.1 for each).

**Figure 6:**
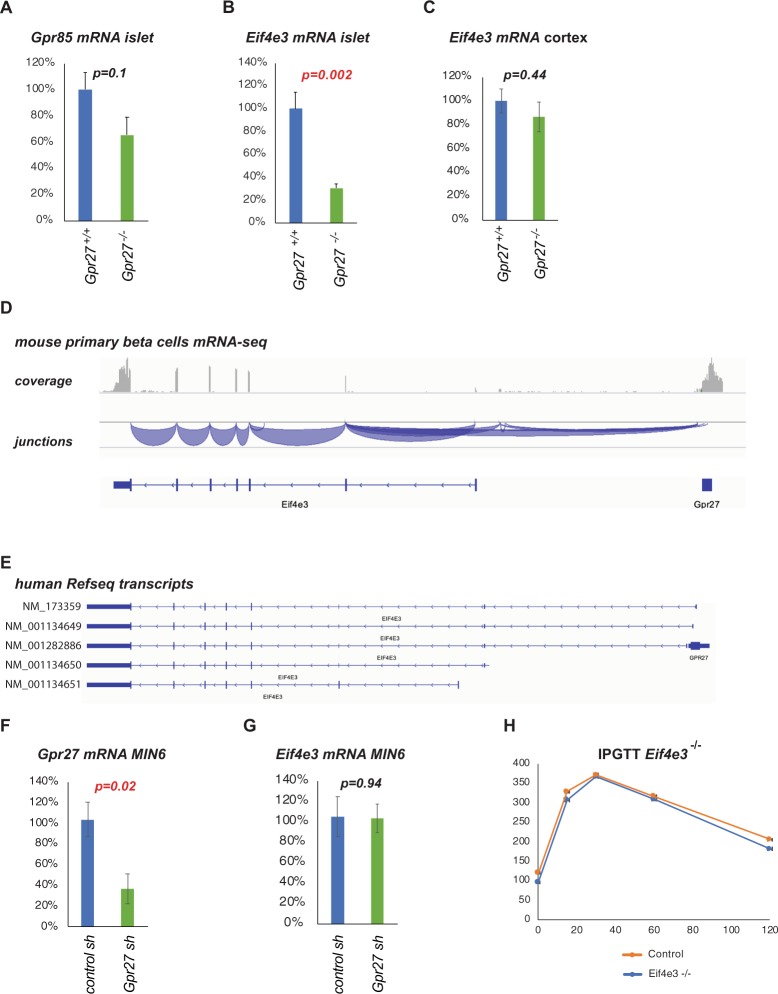
*Gpr27* knockout islets have reduced *Eif4e3* mRNA. (**A**) As in Fig. 1B but for *Gpr85* mRNA. (**B**) As in 6A except for *Eif4e3* mRNA. (**C**) As in Fig. 1C except for *Eif4e3* mRNA. (**D**) mRNA-seq coverage and junctions from sorted primary mouse beta cells showing splicing between the *Gpr27* coding exon and the *Eif4e3* gene. Data plotted from^16^ using^17^. (**E**) Human Refseq *EIF4E3* transcripts showing two that contain overlap with the *GPR27* coding exon. Note the similar splicing structure to 6D. (**F**) MIN6 cells were infected with the indicated adenoviruses. Expression of *Gpr27* was measured by RT-qPCR. (**G**) As in E, but *Eif4e3* was measured. For F and G, n = 4. p values were determined by Student’s t-test. (**H**) IPGTT of 13-week old *Eif4e3* knockout mice versus control mice. n = 8 male knockout mice vs 320 control mice. Error bars indicate standard error. Data plotted from IMPC^6^.

Given the mild phenotype of the *Gpr27* knockout mice, we measured the expression of the two other *Gpr27* family members, *Gpr85* (SREB2) and *Gpr173* (SREB3). Expression of *Gpr85* in islets was unchanged in the *Gpr27* knockout mice (Fig. 6A) while *Gpr173* was not reliably detected above background in either genotype (not shown). We also considered the possibility that the deletion of the *Gpr27* coding exon could have affected neighboring gene expression. While the *Prok2* (the nearest telomeric gene) is not expressed in mouse islets, the nearest centromeric gene, *Eif4e3* is expressed. Surprisingly, mRNA levels of *Eif4e3* were reduced in *Gpr27* knockout islets (Fig. 6B) but not in *Gpr27* knockout cerebral cortex (Fig. 6C). To attempt to explain this, we examined mRNA-seq data from wild type primary mouse beta cells and observed splicing events between the antisense strand of the *Gpr27* exon and the *Eif4e3* gene (Fig. 6D). We observed similar splice junctions in 2 human RefSeq *EIF4E3* transcripts (NM_173359 and NM_001134649), each beginning with an exon located on the anti-sense strand of the *GPR27* coding exon. Taken together, these data suggest that the deletion of the *Gpr27* exon has the unintended consequence of removing the first exon of a subset of *Eif4e3* transcripts. A less likely possibility would be that *Gpr27* mRNA expression is required for *Eif4e3* transcription. To test this, we knocked down *Gpr27* mRNA using an shRNA in MIN6 cells (Fig. 6E). We found that *Gpr27* knockdown did not reduce *Eif4e3* mRNA (Fig. 6F), suggesting that the reduction of *Eif4e3* in *Gpr27* knockout islets is likely due to the deletion of the *Gpr27* exon itself.

Finally, we examined data from the International Mouse Phenotyping Consortium on *Eif4e3* knockout mice to ask if downregulation of *Eif4e3* could partially explain the modestly worsened glucose tolerance of *Gpr27* knockout animals. *Eif4e3* knockout mice had a trend to lower plasma glucose that did not meet their significance threshold (28.38 mmol/L vs 25.17 mmol/L in males, 25.05 mmol/L vs 17.95 mmol/L in females, combined p-value = 0.007)^6^ and an unchanged glucose tolerance (Fig. 6G). On the other hand, the *Eif4e3* knockout male mice had lower body weights (39.8 grams vs 34.0 grams, p = 0.000928 in males)^6^, suggesting that *Eif4e3* downregulation might explain lower body weights in the *Gpr27* knockout mice.

## Discussion

Herein we provide a description of the *in vivo* consequences of *Gpr27* deletion. We found that *Gpr27* loss reduces insulin mRNA *in vivo*, confirming our previous findings *in vitro* in MIN6 cells^5^. This reduced insulin transcript may be caused by a reduction in mRNA levels of *Pdx1* as we found in MIN6 cells after knockdown of *Gpr27*. However, we found only a non-significant trend towards reduction in levels of *Hnf4a* and *Glis3* in *Gpr27* knockout islets in contrast to the clear reductions in these genes that we found in *Gpr27* knockdown MIN6. One possible reason for this difference is that our experiments in cultured cells silenced *Gpr27* for only 72 hours^5^ while the *Gpr27* knockout beta cells have been without the gene for months and may have compensated for the loss of *Gpr27*. Alternatively, this could be due to differences between cultured beta cells and primary beta cells.

We were initially surprised to find that mRNA transcripts of *Eif4e3*, a gene 25 kb away from *Gpr27*, were reduced in islets but not cerebral cortex of *Gpr27* knockout animals. We believe that the most likely reason for this is that unannotated *Eif4e3* transcriptional start sites are located on the antisense strand of the *Gpr27* coding exon. Although the mouse Refseq annotation does not describe such transcripts, we find evidence of them in mRNA-seq data from mouse beta cells. Confirming this, we find very similar overlapping *GPR27/EIF4E3* transcripts are present in the human Refseq annotation. We speculate that these alternative transcriptional start sites  of *Eif4e3* may be more specific to the islet in mouse versus human, possibly explaining why *Eif4e3* levels are unchanged in the brain of *Gpr27* knockout mice and why the mouse Refseq annotation lacks these transcripts (as islet expression data may not have been included). Our observations suggest that future attempts to manipulate the *Gpr27* exon (e.g. by a Cre-lox strategy or CRISPR) must be done with caution since they may also affect *Eif4e3*. Notably, the alternative transcription start sites change only the 5′ untranslated region of *Eif4e3*, suggesting that the primary effect would be on the *Eif4e3* expression level and not on the protein sequence.

We cannot completely rule of the possibility that the phenotype of our *Gpr27* knockout mice is due to local downregulation of *Eif4e3* by the knockout allele. However, we do not believe this is likely since silencing (by RNA interference) of *Gpr27* does not affect *Eif4e3* transcription but does reduce insulin and Pdx1 mRNA^5^ and *Eif4e3* knockout mice have unchanged glucose tolerance.

While we did observe worsened glucose tolerance in the *Gpr27* knockout mice on a chow diet, the p-values were close to our predetermined statistical threshold and the magnitude of the phenotype was quite mild and likely biologically inconsequential. To minimize the chance of a type 1 error, we confirmed worsened glucose tolerance on an independent cohort of mice fed a high fat diet. In both cases, worsened glucose tolerance in combination with reduced plasma insulin after glucose challenge and unchanged insulin tolerance suggest the *Gpr27* global knockout mice have an insulin secretory defect. However, we did not observe a defect in insulin secretion in *ex vivo* islets from *Gpr27* knockout mice. We speculate that one possibility for the normal *ex vivo* GSIS could be the lack of a hypothetical *Gpr27* ligand *ex vivo*. Alternatively, the defect in GSIS in *Gpr27* deficient islets could be below our limit of detection, especially given the modest effect on glucose tolerance. Finally, given *Gpr27’s* expression in the brain, we cannot rule out a primary brain effect of *Gpr27* loss that then causes reduced GSIS in the periphery.

*Gpr27* remains an orphan GPCR. One possible explanation for the phenotype in Gpr27 knockout mice is that there are as of yet unidentified endogenous ligands that stimulate Gpr27, and loss of Gpr27 blocks these signals. Alternatively, Gpr27 may simply have basal activity that is required for insulin production and insulin secretion^7^. Several groups have now described small molecules that may activate *Gpr27*. Through a chemical screen, Dupuis and Hanson identified two small molecules that can trigger *Gpr27* association with β arrestin-2 in 293T cells^8^. Plasmalogens, a form of glycerophospholipid, may also signal through *Gpr27*, although direct binding of plasmalogens to Gpr27 has not been demonstrated^9^. Additionally, two inverse agonists of *Gpr27* have been described^10^. The *Gpr27* knockout mouse will be a useful resource for testing the specificity of these putative ligands.

The G-protein coupling of *Gpr27* has been controversial. While our initial study showed that *Gpr27* over-expression in 293T cells increased IP1 suggesting Gαq coupling, this finding was not reproduced by Dupuis and Hanson who found no evidence of Gαq, Gαs, or Gαi coupling^8^. On the other hand, Martin and Aronstam found evidence of constitutive Gαi coupling in 293T cells^7^. Thus, it is not clear how *Gpr27* signals even in these heterologous over-expression systems. However, impaired insulin secretion in *Gpr27* knockout mice implies that Gpr27 might normally signal to enhance insulin secretion, which would implicate either Gαs or Gαq^11^. Additional studies are needed to further interrogate the signaling mechanism and pathway of *Gpr27*.

In summary, we describe a whole-body knockout mouse for *Gpr27* and show reduced insulin mRNA but only modest effects on glucose homeostasis and an unexpected effect on *Eif4e3* gene expression.

## Methods

### Animals

129SvEvBrd embryonic stem cells were transfected with a targeting vector containing 5′ and 3′ homology arms to the *Gpr27* locus where the entire coding sequence of *Gpr27* was replaced with an IRES-lacZ-neomycin and a bovine growth hormone polyadenylation signal followed by the 3-phosphoglycerate kinase promoter driving a puromycin resistance gene and another bovine growth hormone polyadenylation signal. Cas9 was not used. A clone containing proper integration by homologous recombinaton was confirmed by PCR and southern blot (Supplemental Fig. 1). This clone was injected into blastocysts^12,13^ and germ line transmission was achieved. We obtained heterozygous mice from the Texas A&M Institute for Genomic Medicine and bred the mice to C57B6J for >5 generations in our mouse facility. The mouse line is currently available from Taconic (https://www.taconic.com/knockout-mouse/gpr27-targeted). The sequence of the targeting vector and the final genomic sequence of the targeted allele are in Supplemental Data.

Mice were group-housed in a colony maintained with a standard 12 h light/dark cycle and given food and water ad libitum. Experiments were conducted according to the Guide for the Care and Use of Laboratory Animals, as adopted by the National Institutes of Health, and with approval of the UCSF Institutional Animal Care Use Committee (IACUC). Genotyping for the mutant allele was performed with the following primers: puroLR = GAACCAGCTGATTACCCTGTTATCCCTAC and Gpr27-R = CGCAAAGGTAATGCCACTTGAGG. Genotyping for the wild type allele was performed with the following primers: Gpr27-F = CTGAAAGGCATTGGTTTGTGAAGC and Gpr27-R = CGCAAAGGTAATGCCACTTGAGG. Male mice age 9–15 weeks were used for all experiments unless otherwise specified in figure legends. For high fat diet, mice were placed on Research Diets D12492 (5.21 Kcal/g, 20% protein, 20% carbohydrates, 60% fat) from 3 weeks until 12 weeks of age. For all other experiments, animals were fed PicoLab Mouse Diet 20 (3.75 Kcal/g, 23.2% protein, 55.2% carbohydrates, 21.6% fat). Intraperitoneal glucose tolerance test, insulin tolerance test, and plasma insulin collection were performed as previously described^14^. Briefly, mice were fasted individually for 16 hours prior to 2 g/kg glucose injection or 0.1 mU/kg insulin. Glucose (Freestyle Lite, Abbott) and insulin (mouse insulin ELISA, Mercodia) were measured from blood or plasma taken from the tail.

### Islet RT-qPCR

Pancreatic islets were isolated as previously described^15^ and cultured overnight in RPMI with 10% fetal bovine serum, 1% penicillin/streptomycin, 25 mM HEPES. Total RNA was extracted by Trizol (ThermoFisher) or Direct-zol RNA MiniPrep Kit (Zymo 11–330), treated with DNase I (Turbo DNase, Ambion), and reverse transcribed with Superscript III (ThermoFisher). Gene expression was calculated utilizing the delta delta CT method relative to Gusb. Probes utilized were: Ins1/2^5^, Gpr27^5^, Glis3 (Mm00443081_m1, ThermoFisher), Hnf4a (Mm01247712_m1, ThermoFisher), Pax6 (Mm00615386_m1, ThermoFisher), Pdx1 (Mm00435565_m1, ThermoFisher), Eif4e3 (Mm01182452_m1, ThermoFisher), Gpr85 (Mm00460767_s1, ThermoFisher), Gpr173 (Mm02620389_s1, ThermoFisher), Gusb^5^.

### *Gpr27* knockdown

Control or *Gpr27* shRNA adenovirus^5^ were used to infect MIN6 cells at an MOI of 50, cultured for 3 days and analyzed for *Gpr27* and *Eif4e3* expression as above.

### *Ex vivo* glucose stimulated insulin secretion (GSIS)

Islets were rested for 24 hours after isolation RPMI (Gibco) with HEPES, penicillin, streptomycin, and glutamine supplementation. For static GSIS, 15–20 islets were hand-picked and equilibrated in Krebs-Ringer Bicarbonate HEPES buffer (KRBH; 137 mM NaCl, 4.7 mM KCl, 1.2 mM KH_2_PO_4_, 1.2 mM MgSO_4_, 2.5 mM CaCl_2_, 25 mM NaHCO_3_, 20 mM HEPES) containing 2.8 mM glucose for 1 hour at 37 °C. Equilibration buffer was then removed and islets were sequentially stimulated with KRBH containing glucose and compounds as indicated in figure panels. Islets were lysed in RIPA buffer for determination of total islet insulin content. Islet lysate protein content was determined using the Pierce 660 Protein Assay with Ionic Detergent Compatibility Reagent (ThermoFisher).

### Immunofluorescence imaging

Pancreata were fixed in Z-FIX (Anatech) and paraffin embedded. Five µm sections were deparaffinized and subjected to antigen retrieval using Citrate unmasking solution (Vector H3300). Primary antibodies used: guinea-pig anti-insulin (1:250, Dako A0564), rabbit anti-glucagon (1:250, Immunostar 20076). Secondary antibodies: anti-guinea pig Alexa Fluor 488 (1:500, ThermoFisher A11073), anti-rabbit Alexa Fluor 555 (1:500, ThermoFisher A31572) Images were captured with a Zeiss Apotome widefield microscope.

### Beta cell mass and distribution of islet size

Pancreatic sections as above were taken at approximately every 100 microns through the whole pancreas, fixed and cut as done for the immunofluorescence except only the guinea-pig anti-insulin antibody was used for the primary antibody at 1:1000. The ABC Kit (Elite EK-6100) and DAB Chromogen System (Dako) were used to develop. A Keyence BZ-X710 at 2X magnification was used to capture images. Beta cell area was measured with photoshop for each section and % beta cell area was measured. Beta cell mass was calculated by the product of % beta cell area * pancreas wet weight.

### Statistical analysis

For IPGTT and plasma insulin, a mixed two-way ANOVA with repeated measures was implemented by SPSS (IBM). For multiple testing of individual time points, we used Benjamini-Hochberg post-hoc testing. For AUC and body weight, we used a two-tailed Student’s t-test.

## Supplementary information

Supplementary information

## Data Availability

All data generated or analyzed during this study are included in this published article.
