# Supplementary material for: Deletion of Gpr27 in vivo reduces insulin mRNA but does not result in diabetes
Source: Sci Rep. 2020 Mar 27;10:5629. doi: 10.1038/s41598-020-62358-4 (PMC7101378; doi:10.1038/s41598-020-62358-4)
Supplement: Supplementary file 1 — Supplementary information [file 41598_2020_62358_MOESM1_ESM.pdf]

Supplemental Data

Deletion of Gpr27 in vivo reduces insulin mRNA but does not result in diabetes

Deeksha G. Chopra, Nicholas Yiv, Thomas G. Hennings, Yaohuan Zhang, and Gregory M. Ku*

A

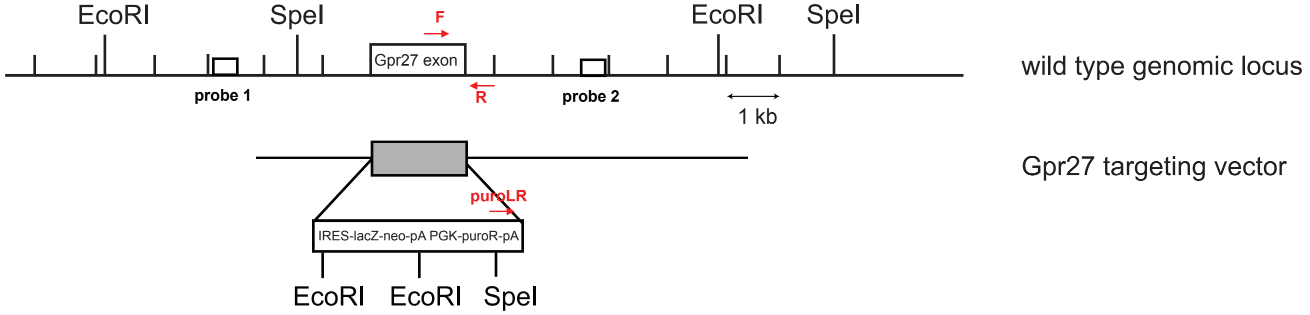

B

**Probe**	**1**	**2**
**Enzyme**	**EcoRI**	**SpeI**
**Wildtype**	**10.6 kb**	**9.2 kb**
**Targeted**	**4.8 kb**	**8.1 kb**

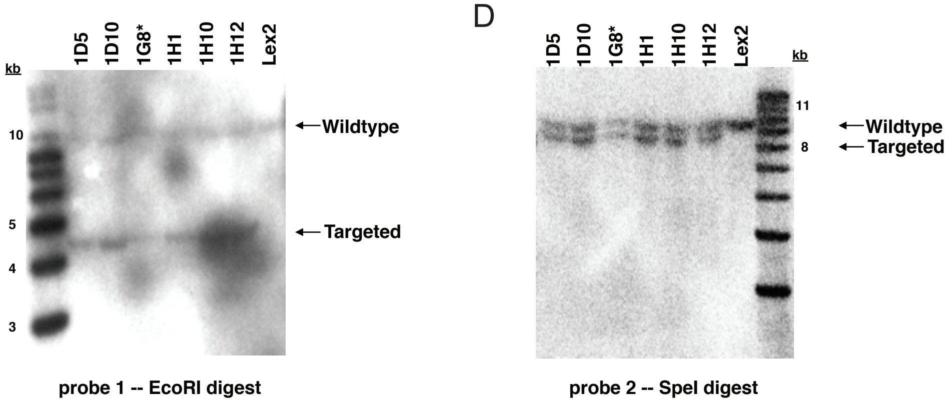

Supplemental Figure 1: Construction of the Gpr27 knockout allele used in this study. (A) (top) Wild type Gpr27 genomic locus showing a single exon and restriction enzyme sites used for southern blot confirmation. Red arrows indicate primers used to detect wild type and mutant alleles. (bottom) Gpr27 targeting vector used to transfect embryonic stem cells for homologous recombination. Small vertical lines placed every 1 kilobase. (B) Predicted southern blot bands after digestion with the indicated enzyme and probed with the indicated probes. Note that probe 1 is external to the targeting construct and probe 2 is internal to the targeting construct. (C) Southern blot of genomic DNA from different ES cell clones using probe 1 following EcoRI digest. Lex2 is the parental embryonic stem (ES) cell line. (D) Southern blot of genomic DNA from ES cells using probe 2 following SpeI digest. Clone 1G8 was the clone was used to generate this line of mice.

Southern probes
19 5' – TATATTCGCGCTGCTTGATCT
20 5' – TCAGCGAGAGAATGGGAAAGG
25 5' – CTGTGTCCCGGATGC
26 5' – CATTGTGGCTGCCGATTT

PCR Genotyping
rev 5' – CGCAAAGGTAATGCCACTTGAGG
f 5' – CTGAAAGGCATTGGTTTGTGAAGC
PuroLR 5' – GAACCAGCTGATTACCCTGTTATCCCTAC

Genomic Sequence Deleted:

GCCGGGCGGCGGCGGCAGCGGCGGAGGGGCCGAGGCTGCCGCGCTGGGCCTTAGGCTGGCCACGCTCAGCCTGCTGCTGTGCGTGAGCCTGGCGGGC
AACGTGCTGTTCGCGCTGCTCATCGTGCGGGAGCGCAGCCTGCACCGCGCGCCGTACTACCTGCTGCTCGACCTGTGCCTGGCCGACGGGCTGCGCGCG
CTCGCCTGCCTCCCGGCCGTCATGCTGGCGGCGCGGCGCGCGGCGGCCGCGGCGGGGACGCCGCCGGGCGCGCTGGGCTGCAAGCTGCTGGCCTTCCT
GGCCGCACTCTTCTGCTTCCACGCGGCCTTCCTGCTGCTGGGCGTGGGCGTCACCCGCTACCTGGCCATCGCGCACCACCGCTTCTACGCCGAGCGCCT
GGCCGGCTGGCCGTGCGCCGCGATGCTGGTGTGCGCCGCCTGGGCGCTGGCTTTGGCCGCGGCCTTCCCGCCGGTGCTGGACGGCGGTGGCGCGGACG
ACGAGGACGCGCCGTGCGCCCTGGAGCAGCGACCCGACGGCGCCCCGGGTGCGCTAGGCTTCCTGCTGCTCCTGGCCGCGGTGGTGGGCGCCACGCAC
CTCGTCTACCTCCGCCTGCTCTTCTTCATCCACGACCGCCGCAAGATGCGACCCGCGCGCCTGGTGCCCGCCGTCAGCCACGACTGGACCTTCCACGGC
CCGGGCGCCACCGGTCAGGCGGCCGCCAACTGGACGGCGGGCTTCGGCCGCGGGCCCACGCCACCTGCGCTCGTGGGCATCAGGCCTGCAGGCCCGG
GCCGCGGCGCCCGGCGCCTCCTGGTGCTGGAGGAGTTCAAGACGGAGAAGAGGCTGTGCAAGATGTTCTACGCCATCACGCTGCTCTTCCTGCTCCTCT
GGGGCCCCTATGTGGTGGCCAGTTACCTGCGCGTCCTGGTGCGCCCGGGAGCTGTCCCGCAGGCCTACCTGACAGCCTCGGTGTGGCTGACATTCGCGC
AGGCCGGCATCAACCCCGTGGTGTGTTTCCTCTTCAACCGGGAGCTGAGGGACTGTTTCCGAGCCCAGTTTCCCTGCTGCCAGAGCCCCCAGGCCACGC
AGGCCACCCTCCCCTGCGACCTGAAAGGCATTGGTTTGTGAAGCGCGCTCTGCC

Selection Cassette: Bgeo/Puro

GGCCATAGCGGCCATTTAAATGGCGCGCCGGATCCGAATTCCTCGAGGCTAGAACTAGCGATAAGCTTCGAGCGGGATCAATTCCGCCCCCCCCCTAA
CGTTACTGGCCGAAGCCGCTTGGAATAAGGCCGGTGTGCGTTTGTCTATATGTTATTTTCCACCATATTGCCGTCTTTTGGCAATGTGAGGGCCCGGAA
ACCTGGCCCTGTCTTCTTGACGAGCATTCCTAGGGGTCTTTCCCCTCTCGCCAAAGGAATGCAAGGTCTGTTGAATGTCGTGAAGGAAGCAGTTCCTCT
GGAAGCTTCTTGAAGACAAACAACGTCTGTAGCGACCCTTTGCAGGCAGCGGAACCCCCCACCTGGCGACAGGTGCCTCTGCGGCCAAAAGCCACGTG
TATAAGATACACCTGCAAAGGCGGCACAACCCCAGTGCCACGTTGTGAGTTGGATAGTTGTGGAAAGAGTCAAATGGCTCTCCTCAAGCGTATTCAAC
AAGGGGCTGAAGGATGCCCAGAAGGTACCCCATTGTATGGGATCTGATCTGGGGCCTCGGTGCACATGCTTTACATGTGTTTAGTCGAGGTTAAAAAA
ACGTCTAGGCCCCCCGAACCACGGGGACGTGGTTTTCCTTTGAAAAACACGATAATACCATGGGGGATCCCGTCGTTTTACAACGTCGTGACTGGGAA
AACCCTGGCGTTACCCAACTTAATCGCCTTGCAGCACATCCCCCTTTCGCCAGCTGGCGTAATAGCGAAGAGGCCCGCACCGATCGCCCTTCCCAACAG
TTGCGCAGCCTGAATGGCGAATGGCGCTTTGCCTGGTTTCCGGCACCAGAAGCGGTGCCGGAAAGCTGGCTGGAGTGCGATCTTCCTGAGGCCGATAC
TGTCGTCGTCCCCTCAAACTGGCAGATGCACGGTTACGATGCGCCCATCTACACCAACGTGACCTATCCCATTACGGTCAATCCGCCGTTTGTTCCCAC
GGAGAATCCGACGGGTTGTTACTCGCTCACATTTAATGTTGATGAAAGCTGGCTACAGGAAGGCCAGACGCGAATTATTTTTGATGGCGTTAACTCGGC
GTTTCATCTGTGGTGCAACGGGCGCTGGGTCGGTTACGGCCAGGACAGTCGTTTGCCGTCTGAATTTGACCTGAGCGCATTTTTACGCGCCGGAGAAAA
CCGCCTCGCGGTGATGGTGCTGCGCTGGAGTGACGGCAGTTATCTGGAAGATCAGGATATGTGGCGGATGAGCGGCATTTTCCGTGACGTCTCGTTGCT
GCATAAACCGACTACACAAATCAGCGATTTCCATGTTGCCACTCGCTTTAATGATGATTTCAGCCGCGCTGTACTGGAGGCTGAAGTTCAGATGTGCGG
CGAGTTGCGTGACTACCTACGGGTAACAGTTTCTTTATGGCAGGGTGAAACGCAGGTCGCCAGCGGCACCGCGCCTTTCGGCGGTGAAATTATCGATG
AGCGTGGTGGTTATGCCGATCGCGTCACACTACGTCTGAACGTCGAAAACCCGAAACTGTGGAGCGCCGAAATCCCGAATCTCTATCGTGCGGTGGTT
GAACTGCACACCGCCGACGGCACGCTGATTGAAGCAGAAGCCTGCGATGTCGGTTTCCGCGAGGTGCGGATTGAAAATGGTCTGCTGCTGCTGAACGG
CAAGCCGTTGCTGATTCGAGGCGTTAACCGTCACGAGCATCATCCTCTGCATGGTCAGGTCATGGATGAGCAGACGATGGTGCAGGATATCCTGCTGAT
GAAGCAGAACAACTTTAACGCCGTGCGCTGTTCGCATTATCCGAACCATCCGCTGTGGTACACGCTGTGCGACCGCTACGGCCTGTATGTGGTGGATGA
AGCCAATATTGAAACCCACGGCATGGTGCCAATGAATCGTCTGACCGATGATCCGCGCTGGCTACCGGCGATGAGCGAACGCGTAACGCGAATGGTGC
AGCGCGATCGTAATCACCCGAGTGTGATCATCTGGTCGCTGGGGAATGAATCAGGCCACGGCGCTAATCACGACGCGCTGTATCGCTGGATCAAATCT
GTCGATCCTTCCCGCCCGGTGCAGTATGAAGGCGGCGGAGCCGACACCACGGCCACCGATATTATTTGCCCGATGTACGCGCGCGTGGATGAAGACCA
GCCCTTCCCGGCTGTGCCGAAATGGTCCATCAAAAAATGGCTTTCGCTACCTGGAGAGACGCGCCCGCTGATCCTTTGCGAATACGCCCACGCGATGG
GTAACAGTCTTGGCGGTTTCGCTAAATACTGGCAGGCGTTTCGTCAGTATCCCCGTTTACAGGGCGGCTTCGTCTGGGACTGGGTGGATCAGTCGCTGA
TTAAATATGATGAAAACGGCAACCCGTGGTCGGCTTACGGCGGTGATTTTGGCGATACGCCGAACGATCGCCAGTTCTGTATGAACGGTCTGGTCTTTG
CCGACCGCACGCCGCATCCAGCGCTGACGGAAGCAAAACACCAGCAGCAGTTTTTCCAGTTCCGTTTATCCGGGCAAACCATCGAAGTGACCAGCGAA
TACCTGTTCCGTCATAGCGATAACGAGCTCCTGCACTGGATGGTGGCGCTGGATGGTAAGCCGCTGGCAAGCGGTGAAGTGCCTCTGGATGTCGCTCCA
CAAGGTAAACAGTTGATTGAACTGCCTGAACTACCGCAGCCGGAGAGCGCCGGGCAACTCTGGCTCACAGTACGCGTAGTGCAACCGAACGCGACCG
CATGGTCAGAAGCCGGGCACATCAGCGCCTGGCAGCAGTGGCGTCTGGCGGAAAACCTCAGTGTGACGCTCCCCGCCGCGTCCCACGCCATCCCGCAT
CTGACCACCAGCGAAATGGATTTTTGCATCGAGCTGGGTAATAAGCGTTGGCAATTTAACCGCCAGTCAGGCTTTCTTTCACAGATGTGGATTGGCGAT
AAAAAACAACTGCTGACGCCGCTGCGCGATCAGTTCACCCGTGCACCGCTGGATAACGACATTGGCGTAAGTGAAGCGACCCGCATTGACCCTAACGC

CTGGGTCGAACGCTGGAAGGCGGCGGGCCATTACCAGGCCGAAGCAGCGTTGTTGCAGTGCACGGCAGATACACTTGCTGATGCGGTGCTGATTACGA
CCGCTCACGCGTGGCAGCATCAGGGGAAAACCTTATTTATCAGCCGGAAAACCTACCGGATTGATGGTAGTGGTCAAATGGCGATTACCGTTGATGTT
GAAGTGGCGAGCGATACACCGCATCCGGCGCGGATTGGCCTGAACTGCCAGCTGGCGCAGGTAGCAGAGCGGGTAAACTGGCTCGGATTAGGGCCGC
AAGAAAACTATCCCGACCGCCTTACTGCCGCCTGTTTTGACCGCTGGGATCTGCCATTGTCAGACATGTATACCCCGTACGTCTTCCCGAGCGAAAACG
GTCTGCGCTGCGGGACGCGCGAATTGAATTATGGCCCACACCAGTGGCGCGGCGACTTCCAGTTCAACATCAGCCGCTACAGTCAACAGCAACTGATG
GAAACCAGCCATCGCCATCTGCTGCACGCGGAAGAAGGCACATGGCTGAATATCGACGGTTTCCATATGGGGATTGGTGGCGACGACTCCTGGAGCCC
GTCAGTATCGGCGGAATTCCAGCTGAGCGCCGGTCGCTACCATTACCAGTTGGTCTGGTGTCAGGGGATCCCCCGGGCTGCAGCCAATATGGGATCGG
CCATTGAACAAGATGGATTGCACGCAGGTTCTCCGGCCGCTTGGGTGGAGAGGCTATTCGGCTATGACTGGGCACAACAGACAATCGGCTGCTCTGAT
GCCGCCGTGTTCCGGCTGTCAGCGCAGGGGCGCCCGGTTCTTTTTGTCAAGACCGACCTGTCCGGTGCCCTGAATGAACTGCAGGACGAGGCAGCGCG
GCTATCGTGGCTGGCCACGACGGGCGTTCCTTGCGCAGCTGTGCTCGACGTTGTCACTGAAGCGGGAAGGGACTGGCTGCTATTGGGCGAAGTGCCGG
GGCAGGATCTCCTGTCATCTCACCTTGCTCCTGCCGAGAAAGTATCCATCATGGCTGATGCAATGCGGCGGCTGCATACGCTTGATCCGGCTACCTGCC
CATTCGACCACCAAGCGAAACATCGCATCGAGCGAGCACGTACTCGGATGGAAGCCGGTCTTGTCGATCAGGATGATCTGGACGAAGAGCATCAGGG
GCTCGCGCCAGCCGAACTGTTCGCCAGGCTCAAGGCGCGCATGCCCGACGGCGAGGATCTCGTCGTGACCCATGGCGATGCCTGCTTGCCGAATATCA
TGGTGGAAAATGGCCGCTTTTCTGGATTCATCGACTGTGGCCGGCTGGGTGTGGCGGACCGCTATCAGGACATAGCGTTGGCTACCCGTGATATTGCTG
AAGAGCTTGGCGGCGAATGGGCTGACCGCTTCCTCGTGCTTTACGGTATCGCCGCTCCCGATTCGCAGCGCATCGCCTTCTATCGCCTTCTTGACGAGTT
CTTCTGAGGGGATCAATTCTCTAGAGCTCGCTGATCAGCCTCGACTGTGCCTTCTAGTTGCCAGCCATCTGTTGTTTGCCCCTCCCCCGTACCTTCCTTG
ACCCTGGAAGGTGCCACTCCCACTGTCCTTTCCTAATAAAATGAGGAAATTGCATCGCATTGTCTGAGTAGGTGTCATTCTATTCTGGGGGGTGGGGTG
GGGCAGGACAGCAAGGGGGAGGATTGGGAAGACAATAGCAGGCATGCTGGGGATGCGGTGGGCTCTATGGCTTCTGAGGCGGAAAGAACCAGCTGG
GGCTCGATCCTCTAGAGTCGAGTACCGGGTAGGGGAGGCGCTTTTCCCAAGGCAGTCTGGAGCATGCGCTTTAGCAGCCCCGCTGGGCACTTGGCGCT
ACACAAGTGGCCTCTGGCCTCGCACACATTCCACATCCACCGGTAGGCGCCAACCGGCTCCGTTCTTTGGTGGCCCCTTCGCGCCACCTTCTACTCCTCC
CCTAGTCAGGAAGTTCCCCCCCGCCCCGCAGCTCGCGTCGTGCAGGACGTGACAAATGGAAGTAGCACGTCTCACTAGTCTCGTGCAGATGGACAGCA
CCGCTGAGCAATGGAAGCGGGTAGGCCTTTGGGGCAGCGGCCAATAGCAGCTTTGCTCCTTCGCTTTCTGGGCTCAGAGGCTGGGAAGGGGTGGGTCC
GGGGGCGGGCTCAGGGGCGGGCTCAGGGGCGGGGCGGGCGCCCGAAGGTCCTCCGGAGGCCCGGCATTCTGCACGCTTCAAAAGCGCACGTCTGCCG
CGCTGTTCTCCTCTTCCTCATCTCCGGGCCTTTCGACCTGCACTGCGCGCCAGCTTACCATGACCGAGTACAAGCCCACGGTGCGCCTCGCCACCCGCG
ACGACGTCCCCAGGGCCGTACGCACCCTCGCCGCCGCGTTCGCCGACTACCCCGCCACGCGCCACACCGTCGATCCAGACCGCCACATCGAGCGGGTC
ACCGAGCTGCAAGAACTCTTCCTCACGCGCGTCGGGCTCGACATCGGCAAGGTGTGGGTCGCGGACGACGGCGCAGCAGTGGCGGTCTGGACCACGCC
GGAGAGCGTCGAAGCGGGGGCGGTGTTCGCCGAGATCGGCCCGCGCATGGCCGAGTTGAGCGGTTCCCGGCTGGCCGCGCAGCAACAGATGGAAGGC
CTCCTGGCGCCGCACCGGCCCAAGGAGCCCGCGTGGTTCCTGGCCACCGTCGGTGTCTCGCCCGACCACCAGGGCAAGGGTCTGGGCAGCGCCGTCGT
GCTCCCCGGAGTGGAGGCGGCCGAGCGCGCCGGGGTGCCCGCCTTCCTGGAGACCTCCGCGCCCCGCAACCTCCCCTTCTACGAGCGGCTCGGCTTCA
CCGTCACCGCCGACGTCGAGGTGCCCGAAGGACCGCGCACCTGGTGCATGACCCGCAAGCCCGGTGCCTGACGCCCGCCCCACGACCCGCAGCGCCCG
ACCGAAAGGAGCGCACGACCCCATGCATCGATGATCTAGAGCTCGCTGATCAGCCTCGACTGTGCCTTCTAGTTGCCAGCCATCTGTTGTTTGCCCCTC
CCCCGTGCCTTCCTTGACCCTGGAAGGTGCCACTCCCACTGTCCTTTCCTAATAAAATGAGGAAATTGCATCGCATTGTCTGAGTAGGTGTCATTCTATT
CTGGGGGGTGGGGTGGGGCAGGACAGCAAGGGGGAGGATTGGGAAGACAATAGCAGGCATGCTGGGGATGCGGTGGGCTCTATGGCTTCTGAGGCGG
AAAGAACCAGCTGATTACCCTGTTATCCCTACTCGACCTCGAGGGCGCGCCATTTAATGGCCAGCGAGGCC

Targeted Locus:

ACGCGCAGCCTGGTCGAACGCAGACGCGTGTTGATGGCCGGGGTACGAAGCCATACGCGCTTCTACAAGGCGCTGGCCGAAGAGGTGCGGGAGTTTC
ACGCCACCAAGATCTGCGGCACGCTGTTGACGCTGTTAAGCGGGTCGCTGCAGGGTCGCTCGGTGTTCGAGGCCACACGCGTCACCTTAATATGCGAA
GTGGACCTCGGACCGCGCCGCCCCGACTGCATCTGCGTGTTCGAATTCGCCAATGACAAGACGCTGGGCGGGGTTTGCTCGACATTGGGTGGAAACAT
TCCAGGCCTGGGTGGAGAGGCTTTTTGCTTCCTCTTGCAAAACCACACTGCTCGACATTGGGTGGAAACATTCCAGGCCTGGGTGGAGAGGCTTTTTGC
TTCCTCTTGAAAACCACACTGCTCGACGGATCCCTATGGATTCATGGTAAATCCATAAAATCAAGGGAAATACAACTACTTCTTATTTATAAACCTAAC
TTCCGACTCTGTCGCTGTTTTGCCCTGCTGGTGGGTTATGTCGTTTCAGGTTTTAGGGCAGTTCCTTGCTTCTTTAAGAGTGCCATAAATCTGGCGCACAT
ATTTTACGATACACGGTCACTCAAGCTAGTGTATGTCAGCATGGAAGCTGGTACTTCATCAGCTGGGGTCAAACTGTTCTCATCTTTAGTCACTGTCCTT
CCCTGAGCTGAGGTATGAGCATCTTGGCTGAGACGTCTATCCACAGTCATCATCTTCAGTGACCACATGTTACCATGTACATACCTTGTGCTTTGCTTTC
TATTAGTAGGTCTCACCCAACCGACTGTTCCTCACAGCAGGCCTTTGAGATTCAGAGGAAACAGAACTTTCCCAGAGGTACACAGATAATGACTGATTT
GGGAATGAGCCAGGTTATATCTAACACCAATGCATTCTTGAAGTTATGGCCGACTGCTACAGAACAGCCCAGTGCTATGCTCTGATAACCCAACATCCC
ATGGGCCCAGACTCTGGCTCGCTGCTTCTCAGAGATATGATGGGGCTGTCAATACAATGTTCTCCAAGACAGAAACCTGAGGCAGAGGTCAAGAACGC
TTCCATGATGATGGAGCCCACAGCCACAGCTGCGTGTGTCAAAATGTGCCAACTCGTCTCTCTGAACAAGCTGGTTTATATCACCAGTCACACTACGTG
CATCAAAGATGTAACATAACGGATGGGAGGGGGGCAATTTCAACTCCTCTATCTATGTACAAAAGAAAAATTACTTACAAATCAAATTTTTTATTGGA
AAAAAAAAAACCTCATTTGCCTTTAACAAGCGATAATCAATTTGGCGCCTGAGAAAATATTTGAAAGTCTTGTTTGAAGTAAAAGGAAAACAAGTTGA
TCCCAGCCAATAAAATGTTAATGACTTTTCTTTAAGTATGAATCTATTTTAATCTATTGAATAAATGCTGAAAATGAAGGCTGTAGACACGTGAGTGCT
GCCACAGTCATGGTTTACATGTCCTGCTGCTGCCGGGCTTATTTGATTCTGGGCAAATGATTAAACCAAATCTCAGATCCCAAAGATAAATGTATCAGA
CCATATAGCAGTCAGTCTGTCTGTCTGTCTGTCATCTACTGTCTATCTAGCCATCATCTATTTGTCTGTCTCTCACAAGGGAACCATGATCTCTTTCTTTC
CCTTCTCTGAAGATTAGCTCTCTCCACTTGGGACACAGACACATTTAAACTCCATTTTTTTTCCCTCACCTACAAACTAAAGACCCAGAGACCCTAGAA
AACCAATACCACATTTTGTATATGTTAACACTCTACATTTTGAGAGGTTAAATGATTTGCTGGGAGATTTATACTCATAACAGCAACGCTATCAACATTC
CAACTCAGTGGGGAAGGGTCTGACCTGAAATAAGAGGACCTGGGCCCTACTTCCCACTCAGCCATAAACGGGTCAGTAAAACCTGAGTGTCTGACTGG
GTGTTTTGAAAAGTTCTTCTCCCTTTAAAGGCACTCCCGTTCTCAGTCCCGAGCACCTGCTACTCACTGCCGGTTTTCCTTCTCGCTGCTTTCTCATCCCA
AGAGGCAGAGGCTGGGGCTGGGGAGATAGATGACTCAGTAAGGTCCTCTGCCTTGCAGAAGGACCTAAGTTTGATCCATAGAACTCATGTTAAAATAG
CCAGCTAAGTTTGCTTGTAACCTCAGTGCCAGGGAAGAGGAGACAGATGACTCTCTGGGGCTAGTTAGCCAGGTAGCCCAGGCTACTTAGCCAGCTCC
ACACCAATAGAAGAGCAGGAAATCCTGTCTCAAGAACCTGAGGAGGCATACTTTAGGTTGTCTTCTGCACCTAACCCCACGAACATGCACACTCTATA
TATTCGCGCTGCTTGATCTGCTGAGTAGAAATTGTAACCATATATACCACACACTGCCGGCCACAGCAACGACATTAAATAACACTTCGTTGTTTATTT
AACACAGTGCTTGGCATCTGGGTGCTCAGTATATGGAAGCTACCTTTCACATCAACATAAAGAACCCAGTGATGGTGTCTTCAAGGACTGTAAGGAATT
TCCTTCTCTGAGCAAGTACGGCCCACATGGTGGCAAAATCTCATTGTCTAGGGCTATGCATCTGGTTTTAAACAAAGAAAAAGATCCCTTTCCCATTCT
CTCGCTGAGAGTGGGAGTCCATCACATTGTGCCTGCTGTAGTGTGGGACTTCTAGAAAGCTCCTTGAAGGGGGAGCCAACCTACATGCCAGGAACACT
CCCCCTCTTCCCCCTCTCTCTCTGCTGGGGACATCTGCAGCCCATCCTGATCTCGAGGGGAGGGGAACACTTCCACAGATCCAGTCTGTGGGCCAACAA
CCAACACCAGCATCCACTCACCTCCATGATGGAAATACAATTCCTAGCCCGTGCACCCTCTCAAACACACTGCTTTTTTGCTGTCACATATATTTATATG
ACTGTCCAATCACCTCCTCTTGACCACGTAGGAGTACCAAGAAAGGCATCCCCTTGAGATTTTTGCGAAAATGTCCAAACTGTCTTCCAAGGCCTTGTT
ACTCAAAGTGGGGCGAACAGGCAATATAAGTGTCACTATCTAGACCCATTCTGGACCTACTGAGTCAGAACTGTGTTTTAAGGGGACCCCCCCCCCCC
CAGTGACTTCCAAATGCAAAGTCTTAGGCTTTGGACTAACATCTAGGTTCTGCGCTCCTGGGCTTTGGCTTGTTAGATGACCCAGGCTGGCTTCCAATTT
GCCACCCCTGACATATCAGCTTCTGGGGTGCTGAGACCACAGGTGCATATGACCACAAGGATGCTGTTATTCTTCTTCAACCCTGCTGCCAATGTAACC
CTGCTTATGTTATAAGCAGTGTGTTGTCCTCACACCTCTCTCCTTCTCCAGCACCTATCACAGGCTAAGTTTTGACTCAGCCCGGTACTTCAGGCTCTCTA
GGATGGTGACTTTAGGAATGCCTACCAAGATGCCCATTTATGCACGTAGAATTTTTAATGGGTATATCTGAAAAACACCACAGTCCTTAATTTTGGTTC
CCATGTTAAGCAATGAGGATAGTGATATAAACACACACACACACACACACACACGCGCGCGCGCACTATTTAGGGACTAGTCAGATGGACAAGATTTT

AAGAAATAAAACCAGATGATGGGAAACTCTTAAGTGCTAGGAAGCAAATAAAGAGGCCAGTGACAGGCAGTCTGCTCCAAAAGCCTTAAGAATAAGG
GCTCATTTGAGAAGCTGGTATGGACAGTGGGCCCAGCAGGAGAAAAGGTGTTTAAGGGCCAGAACATGGGACTGGCCATTTCAGACTAACCCAGGGA
ATGGATAGATATCCCAGGAAAGACACACATTATTTAAGGAAAACGCCTGATTTCTTTCTGTGGTACACGTTTAGTTCCACTTAAGAGTTAGAGAGCAAG
CACGCTTGAGGGTCCCTGAAAGTCCAGCCTTGGAAACAGGACTAAGTAAAACTGAGATTCCAGCTGGGCCACTTCCTGGCTCTGGACTCCCTTGCCCTC
TAAACCTAGGCTTGCTGGGGTAACTGGTAGGCTTCCGAATAGAAAGACAACTTTACAGTTGTCTCTGGGGGTAAGGGTGAAGGGGGTACACTACCCCC
CTCCCCGCGTGCTACCGCCCACATTAGGCTTCACCTGGGTATCTGTGTTCTGTGCTCACCCTGTTATGTTGCTGCCCATCATTACCAGCTTTCAAACAGA
AGGTTCATCCCAGACATCCCTGCCTCCTCTCCCCCCAGGCCTCCCAGACCCTGCCACCCCACCGTGCCCCCAGCTCCCGGCCACAGTGACTCCAGCGCT
GGCGCTTGACGCCGCCGCCGCCGGACCCCCGTTTACCTGGGGCCAAACGCGACTTGTCGCCACCTCGCGTCCTGGGCCCTGCCTCCCCTCCCGGCTGTC
GCAACGGCGCCCCCAGCCCAGTCAGGCCCGGTGCGGCGCGGGATGCCTCGGCGGGACTGGCGGCGGCGGCCACGCTGCGCGCTCCGGCGGAGGGGAG
GGGGCTCGGCGGCGGCGGCGAAGGCGGTGGCGAAGGCGGCGGCAGCGGCAGCGGCAGCGGCGGGCCCGGCGGGGGGCAGAGCCAAGAGGCGCGGC
GGCGGAGGAGGAACGGGAGGAGGACGCGGAGCATGCGGGGGGCTGCAGGCCTGCCCGCAGTGAAGCCCGCCGCGCCCAGGGCGCAGGGAGTGGCCG
TCGCCTCCTGCGCGCACCGCGACTGAGATGGCGGCGGCGGCGGCGCGGCCGAGGCGCGCCGAGCCCCCGGGGCAGGGCCGGCCGGGCGCGGGCTGAC
AACCCCGCGGGCCGGGAGAGCTAGCGTGGAGGAGCGCAGGCCCCGGGGCGGCCTACGGCGAGCGGCGAGGGCCGGCGGGGAGGAGGAGGAGCACG
GCCGCGATGGCGACGCTAGTGACGCTCTAGAGGCCATAGCGGCCATTTAAATGGCGCGCCGGATCCGAATTCCTCGAGGCTAGAACTAGCGATAAGCT
TCGAGCGGGATCAATTCCGCCCCCCCCCTAACGTTACTGGCCGAAGCCGCTTGGAATAAGGCCGGTGTGCGTTTGTCTATATGTTATTTTCCACCATATT
GCCGTCTTTTGGCAATGTGAGGGCCCGGAAACCTGGCCCTGTCTTCTTGACGAGCATTCCTAGGGGTCTTTCCCCTCTCGCCAAAGGAATGCAAGGTCT
GTTGAATGTCGTGAAGGAAGCAGTTCCTCTGGAAGCTTCTTGAAGACAAACAACGTCTGTAGCGACCCTTTGCAGGCAGCGGAACCCCCCACCTGGCG
ACAGGTGCCTCTGCGGCCAAAAGCCACGTGTATAAGATACACCTGCAAAGGCGGCACAACCCCAGTGCCACGTTGTGAGTTGGATAGTTGTGGAAAGA
GTCAAATGGCTCTCCTCAAGCGTATTCAACAAGGGGCTGAAGGATGCCCAGAAGGTACCCCATTGTATGGGATCTGATCTGGGGCCTCGGTGCACATG
CTTTACATGTGTTTAGTCGAGGTTAAAAAAACGTCTAGGCCCCCCGAACCACGGGGACGTGGTTTTCCTTTGAAAAACACGATAATACCATGGGGGATC
CCGTCGTTTTACAACGTCGTGACTGGGAAAACCCTGGCGTTACCCAACTTAATCGCCTTGCAGCACATCCCCCTTTCGCCAGCTGGCGTAATAGCGAAG
AGGCCCGCACCGATCGCCCTTCCCAACAGTTGCGCAGCCTGAATGGCGAATGGCGCTTTGCCTGGTTTCCGGCACCAGAAGCGGTGCCGGAAAGCTGG
CTGGAGTGCGATCTTCCTGAGGCCGATACTGTCGTCGTCCCCTCAAACTGGCAGATGCACGGTTACGATGCGCCCATCTACACCAACGTGACCTATCCC
ATTACGGTCAATCCGCCGTTTGTTCCCACGGAGAATCCGACGGGTTGTTACTCGCTCACATTTAATGTTGATGAAAGCTGGCTACAGGAAGGCCAGACG
CGAATTATTTTTGATGGCGTTAACTCGGCGTTTCATCTGTGGTGCAACGGGCGCTGGGTCGGTTACGGCCAGGACAGTCGTTTGCCGTCTGAATTTGAC
CTGAGCGCATTTTTACGCGCCGGAGAAAACCGCCTCGCGGTGATGGTGCTGCGCTGGAGTGACGGCAGTTATCTGGAAGATCAGGATATGTGGCGGAT
GAGCGGCATTTTCCGTGACGTCTCGTTGCTGCATAAACCGACTACACAAATCAGCGATTTCCATGTTGCCACTCGCTTTAATGATGATTTCAGCCGCGCT
GTACTGGAGGCTGAAGTTCAGATGTGCGGCGAGTTGCGTGACTACCTACGGGTAACAGTTTCTTTATGGCAGGGTGAAACGCAGGTCGCCAGCGGCAC
CGCGCCTTTCGGCGGTGAAATTATCGATGAGCGTGGTGGTTATGCCGATCGCGTCACACTACGTCTGAACGTCGAAAACCCGAAACTGTGGAGCGCCG
AAATCCCGAATCTCTATCGTGCGGTGGTTGAACTGCACACCGCCGACGGCACGCTGATTGAAGCAGAAGCCTGCGATGTCGGTTTCCGCGAGGTGCGG
ATTGAAAATGGTCTGCTGCTGCTGAACGGCAAGCCGTTGCTGATTCGAGGCGTTAACCGTCACGAGCATCATCCTCTGCATGGTCAGGTCATGGATGAG
CAGACGATGGTGCAGGATATCCTGCTGATGAAGCAGAACAACTTTAACGCCGTGCGCTGTTCGCATTATCCGAACCATCCGCTGTGGTACACGCTGTGC
GACCGCTACGGCCTGTATGTGGTGGATGAAGCCAATATTGAAACCCACGGCATGGTGCCAATGAATCGTCTGACCGATGATCCGCGCTGGCTACCGGC
GATGAGCGAACGCGTAACGCGAATGGTGCAGCGCGATCGTAATCACCCGAGTGTGATCATCTGGTCGCTGGGGAATGAATCAGGCCACGGCGCTAATC
ACGACGCGCTGTATCGCTGGATCAAATCTGTCGATCCTTCCCGCCCGGTGCAGTATGAAGGCGGCGGAGCCGACACCACGGCCACCGATATTATTTGC
CCGATGTACGCGCGCGTGGATGAAGACCAGCCCTTCCCGGCTGTGCCGAAATGGTCCATCAAAAAATGGCTTTCGCTACCTGGAGAGACGCGCCCGCT
GATCCTTTGCGAATACGCCCACGCGATGGGTAACAGTCTTGGCGGTTTCGCTAAATACTGGCAGGCGTTTCGTCAGTATCCCCGTTTACAGGGCGGCTT
CGTCTGGGACTGGGTGGATCAGTCGCTGATTAAATATGATGAAAACGGCAACCCGTGGTCGGCTTACGGCGGTGATTTTGGCGATACGCCGAACGATC
GCCAGTTCTGTATGAACGGTCTGGTCTTTGCCGACCGCACGCCGCATCCAGCGCTGACGGAAGCAAAACACCAGCAGCAGTTTTTCCAGTTCCGTTTAT
CCGGGCAAACCATCGAAGTGACCAGCGAATACCTGTTCCGTCATAGCGATAACGAGCTCCTGCACTGGATGGTGGCGCTGGATGGTAAGCCGCTGGCA
AGCGGTGAAGTGCCTCTGGATGTCGCTCCACAAGGTAAACAGTTGATTGAACTGCCTGAACTACCGCAGCCGGAGAGCGCCGGGCAACTCTGGCTCAC
AGTACGCGTAGTGCAACCGAACGCGACCGCATGGTCAGAAGCCGGGCACATCAGCGCCTGGCAGCAGTGGCGTCTGGCGGAAAACCTCAGTGTGACG
CTCCCCGCCGCGTCCCACGCCATCCCGCATCTGACCACCAGCGAAATGGATTTTTGCATCGAGCTGGGTAATAAGCGTTGGCAATTTAACCGCCAGTCA
GGCTTTCTTTCACAGATGTGGATTGGCGATAAAAAACAACTGCTGACGCCGCTGCGCGATCAGTTCACCCGTGCACCGCTGGATAACGACATTGGCGTA
AGTGAAGCGACCCGCATTGACCCTAACGCCTGGGTCGAACGCTGGAAGGCGGCGGGCCATTACCAGGCCGAAGCAGCGTTGTTGCAGTGCACGGCAG
ATACACTTGCTGATGCGGTGCTGATTACGACCGCTCACGCGTGGCAGCATCAGGGGAAAACCTTATTTATCAGCCGGAAAACCTACCGGATTGATGGT
AGTGGTCAAATGGCGATTACCGTTGATGTTGAAGTGGCGAGCGATACACCGCATCCGGCGCGGATTGGCCTGAACTGCCAGCTGGCGCAGGTAGCAGA
GCGGGTAAACTGGCTCGGATTAGGGCCGCAAGAAAACTATCCCGACCGCCTTACTGCCGCCTGTTTTGACCGCTGGGATCTGCCATTGTCAGACATGTA
TACCCCGTACGTCTTCCCGAGCGAAAACGGTCTGCGCTGCGGGACGCGCGAATTGAATTATGGCCCACACCAGTGGCGCGGCGACTTCCAGTTCAACA
TCAGCCGCTACAGTCAACAGCAACTGATGGAAACCAGCCATCGCCATCTGCTGCACGCGGAAGAAGGCACATGGCTGAATATCGACGGTTTCCATATG
GGGATTGGTGGCGACGACTCCTGGAGCCCGTCAGTATCGGCGGAATTCCAGCTGAGCGCCGGTCGCTACCATTACCAGTTGGTCTGGTGTCAGGGGAT
CCCCCGGGCTGCAGCCAATATGGGATCGGCCATTGAACAAGATGGATTGCACGCAGGTTCTCCGGCCGCTTGGGTGGAGAGGCTATTCGGCTATGACT
GGGCACAACAGACAATCGGCTGCTCTGATGCCGCCGTGTTCCGGCTGTCAGCGCAGGGGCGCCCGGTTCTTTTTGTCAAGACCGACCTGTCCGGTGCCC
TGAATGAACTGCAGGACGAGGCAGCGCGGCTATCGTGGCTGGCCACGACGGGCGTTCCTTGCGCAGCTGTGCTCGACGTTGTCACTGAAGCGGGAAGG
GACTGGCTGCTATTGGGCGAAGTGCCGGGGCAGGATCTCCTGTCATCTCACCTTGCTCCTGCCGAGAAAGTATCCATCATGGCTGATGCAATGCGGCGG
CTGCATACGCTTGATCCGGCTACCTGCCCATTCGACCACCAAGCGAAACATCGCATCGAGCGAGCACGTACTCGGATGGAAGCCGGTCTTGTCGATCA
GGATGATCTGGACGAAGAGCATCAGGGGCTCGCGCCAGCCGAACTGTTCGCCAGGCTCAAGGCGCGCATGCCCGACGGCGAGGATCTCGTCGTGACC
CATGGCGATGCCTGCTTGCCGAATATCATGGTGGAAAATGGCCGCTTTTCTGGATTCATCGACTGTGGCCGGCTGGGTGTGGCGGACCGCTATCAGGAC
ATAGCGTTGGCTACCCGTGATATTGCTGAAGAGCTTGGCGGCGAATGGGCTGACCGCTTCCTCGTGCTTTACGGTATCGCCGCTCCCGATTCGCAGCGC
ATCGCCTTCTATCGCCTTCTTGACGAGTTCTTCTGAGGGGATCAATTCTCTAGAGCTCGCTGATCAGCCTCGACTGTGCCTTCTAGTTGCCAGCCATCTG
TTGTTTGCCCCTCCCCCGTACCTTCCTTGACCCTGGAAGGTGCCACTCCCACTGTCCTTTCCTAATAAAATGAGGAAATTGCATCGCATTGTCTGAGTAG
GTGTCATTCTATTCTGGGGGGTGGGGTGGGGCAGGACAGCAAGGGGGAGGATTGGGAAGACAATAGCAGGCATGCTGGGGATGCGGTGGGCTCTATG
GCTTCTGAGGCGGAAAGAACCAGCTGGGGCTCGATCCTCTAGAGTCGAGTACCGGGTAGGGGAGGCGCTTTTCCCAAGGCAGTCTGGAGCATGCGCTT
TAGCAGCCCCGCTGGGCACTTGGCGCTACACAAGTGGCCTCTGGCCTCGCACACATTCCACATCCACCGGTAGGCGCCAACCGGCTCCGTTCTTTGGTG
GCCCCTTCGCGCCACCTTCTACTCCTCCCCTAGTCAGGAAGTTCCCCCCCGCCCCGCAGCTCGCGTCGTGCAGGACGTGACAAATGGAAGTAGCACGTC
TCACTAGTCTCGTGCAGATGGACAGCACCGCTGAGCAATGGAAGCGGGTAGGCCTTTGGGGCAGCGGCCAATAGCAGCTTTGCTCCTTCGCTTTCTGGG
CTCAGAGGCTGGGAAGGGGTGGGTCCGGGGGCGGGCTCAGGGGCGGGCTCAGGGGCGGGGCGGGCGCCCGAAGGTCCTCCGGAGGCCCGGCATTCTG
CACGCTTCAAAAGCGCACGTCTGCCGCGCTGTTCTCCTCTTCCTCATCTCCGGGCCTTTCGACCTGCACTGCGCGCCAGCTTACCATGACCGAGTACAA
GCCCACGGTGCGCCTCGCCACCCGCGACGACGTCCCCAGGGCCGTACGCACCCTCGCCGCCGCGTTCGCCGACTACCCCGCCACGCGCCACACCGTCG
ATCCAGACCGCCACATCGAGCGGGTCACCGAGCTGCAAGAACTCTTCCTCACGCGCGTCGGGCTCGACATCGGCAAGGTGTGGGTCGCGGACGACGGC
GCAGCAGTGGCGGTCTGGACCACGCCGGAGAGCGTCGAAGCGGGGGCGGTGTTCGCCGAGATCGGCCCGCGCATGGCCGAGTTGAGCGGTTCCCGGC
TGGCCGCGCAGCAACAGATGGAAGGCCTCCTGGCGCCGCACCGGCCCAAGGAGCCCGCGTGGTTCCTGGCCACCGTCGGTGTCTCGCCCGACCACCAG
GGCAAGGGTCTGGGCAGCGCCGTCGTGCTCCCCGGAGTGGAGGCGGCCGAGCGCGCCGGGGTGCCCGCCTTCCTGGAGACCTCCGCGCCCCGCAACCT
CCCCTTCTACGAGCGGCTCGGCTTCACCGTCACCGCCGACGTCGAGGTGCCCGAAGGACCGCGCACCTGGTGCATGACCCGCAAGCCCGGTGCCTGAC

GCCCGCCCCACGACCCGCAGCGCCCGACCGAAAGGAGCGCACGACCCCATGCATCGATGATCTAGAGCTCGCTGATCAGCCTCGACTGTGCCTTCTAG
TTGCCAGCCATCTGTTGTTTGCCCCTCCCCCGTGCCTTCCTTGACCCTGGAAGGTGCCACTCCCACTGTCCTTTCCTAATAAAATGAGGAAATTGCATCG
CATTGTCTGAGTAGGTGTCATTCTATTCTGGGGGGTGGGGTGGGGCAGGACAGCAAGGGGGAGGATTGGGAAGACAATAGCAGGCATGCTGGGGATG
CGGTGGGCTCTATGGCTTCTGAGGCGGAAAGAACCAGCTGATTACCCTGTTATCCCTACTCGACCTCGAGGGCGCGCCATTTAATGGCCAGCGAGGCC
GGTACCCAATTCGCCCTATAGACCCAGGCTCCCTCTGGCTTCGATTGTGACCGCTCATCTTTCCCTTCTGTCCCGTTGTCTGGTTTTTCTCTGCTGCCTTC
AGGAGACTCTGAAAGTGGACACGCACTTGGATTGTATAGACCCCTGTTCTGGGGCCGGGGGAGGGATGCTGAGGTCCCGTTCTGTCCTTTAATTATACT
TTCTTCCTGACAGTAGGCCCTGCAGTCTTTTGTACTGGTACCGACTTCTTTCTTCCACGTGTGATTTTTTTTTTTTTTTAAATAAAGGCTACACTGGTTT
TATTCATGCAAAGTTTCCTAGAGACCATGGCCAGTTTTCTACAGAGGCTATTTTTGACAACCTCAAGTGGCATTACCTTTGCGTTGCAGTGAAGGAGAG
CGAGCTAGGAGATGTTGGAGATGGAAGAGAGGGACAGAGTTGTCTAAAGTCCCCTCAGAACTGCAGGCCACAGGGCCAGGTGAGCTGTGTGCCTTGTT
TGGGGGGTGTGGGGGACTTCTCCCAGTGGTGACACTGAACTTTAGGAGAAAATGCGAATGGGTAGGAGGACGGTCTTATTTGAGTCACTTAAGGAGCA
AGGTCTGGGGCCCTGTGTAAGTTATTATAAAACAATGCATCAGCAAACAGCTGCTCTGTAGACCAGTCTTGGATGGAAACTTTCTGTGAGACTGTGACT
TTTAAACAGTAAAAAGTGTTTTGGATAGTAATGTGGAGAAAAAAAAAACAGTATTGCATGTGTTTGTACCGATTGTGATTTGCGGATGGGGTGGGCTCT
GGAGGGCAGAAAAATACAGGTTTGTTTGGGTTAGGGGTTTGGGGGATTTTCCCCCCCAATAAAAAGTTAAAATACTTTTGTGGAAATGGCTTGTGCTTT
GATAAGCCCAGTTTTACTCTGTATTATGAGCTAATTTTCCCTCTGAAGACTTGTCTGTGGTTAAGAAGATATGGGATATAACGGTGCCTCTCCCAGGAG
ACAAGCATTTTCCTGCGGTGTTTGCTAAGTTAGGTGGTGCTGTTCTACTCATGGCTGTTTTCAATGATTAATAAGAAACCATAATTTAAAATACGATCTC
TTTGTTAGACATTGTCACTTGACACCGAAGGACTTCATTGCAAGTACTGTATAATACTTTTGCTTAAACTGCTCTGTTTTTATTAATGTAACTTGCGTTT
TGAAGATTTCTATCTGTACTTCTCTTTGCATTACACAAATATACCAGTATTTTCACTCTGGAGTGGCTGTTTTGTTTTTGAGGCCAAATCTCTTAGGTCTG
TTGCTAAGTAACACATGCTGTCCGTTGGTGTCTGCTGTCCCGTGTCAGGTGTTTGGTTTGGATATTGGCATCTCTTGTGAACAAGGGTGGGGGTTAGTCA
TGGAGGCCAGACGCTGAGCTCTGTGGCTCTTCCTCTCCATGTTATGTTTTTGAGACAGGGTCTCTCCGTCAAACAGAAGCTTGCCAATTGTCTGGGCTGG
CTGGCCAGGAAGCCCGGGGACCTTAAGCCTGGGAATTTCAGGTGCCCCCAGATTTTTTCTTGGAGCTTAGCCTCTCAAGCTTCTGCAGCAAGCAGGTCA
CCATTGAGCCATCTCCTAAGCATGGGCTACAAATGGAGCTCAGGAGGAGAGCATTTGCCTAGAACCTGAGAGGCCTGGAGTTTGATTCCTAGAGTTGC
CGGGGGTGGGGGGTAATAAACTCCACTGGTTTAAAAGAAAAAAAAAATCAATTTGACAAACTATTCATAATGGATGTAGTAAATTTATGGTTTATTTTG
AGGATAACATTAACTATTAAACATTATGTTCAAGAGAGATGAACAATAAGGGCGTGTGGCCAATCTGTCTCCACTAAATAGCATCATTTTGTTCGGCTT
GTGAGAGCTGAAGACACCAGTCCACTTGGAAGGCTCCTGTTCTCCCCGTGGCCTCAGTTCAGGTAGAGTTCCACCCCTGGGTTTTCAAGGGTCATTCAT
CAGAAATCTTAAGTCCAAGACAGAGAGGGCAGGGGAAATTAATCCTCCTCTCTCACATGCCTATCCAAGTGACTCAAGTTCAAGTCACAGAGGTCACC
AAGGCCATGTTTTATTCAGAATAATTGGCACCCAGCATGCCACAGGAATCCCTCCATCCCAGGATATCCCTGAGTTATTTTGATCTTGGGCAGCAGCCA
GACTCTATCAGAGACACATCACCAGGTGATGCTGGGACTCCTGAGTCCCAGGGTTATTTTCAGAGGAATTAGATGGCCGGGCTTACACAGCTCAGTGG
TTCCTATTGGGTTGTGCTGAGTTGGGAGCTCTTTTCCACGGAGAAGTCACCTTCTACATCCCTCAGTTGGCTCAAGTCGTGATTTACTTTCGACATGCTG
TGTACAAACTTGCTTATAGAGGAAGGCATGGTTTCCACTGAGAATGCAAGGCAGAGCAACAAGCAGCCATTAAGGACTGGGCAGCTGTGTCCCGGATG
CTCAGCTCATTATAGCAGAGCAGGTACAGTGCACTCAAAGAAGACAGGAACTAAGTAAGATGGCTCATGTGCTAATCTGGGATATTAATTCAGTCTGC
CCCATCCCCAGGGTGAGATGAATGAGTTGTCCCCATGCTCAAAGCAAAAGAAGGGGAAAAGCTATGTATAGAAATCTCTATCTTCATGTGCTGTCATG
GATTTATTGAGTTTAAAATAAGCAGTTTCTGGTCTTTGGTACCTTCTATCTAGTAGCTTTGCAGCAAGAGTCGTGCTGTGTAAATTGAAACCTGACTTTC
CCTTTTTAAAAAAGTGGGTACTTCTAGCAAGTTGGGGAGCACATAACAAATCGGCAGCCACAATGACAGGCTCTGGACCCACCTTGCAGTCTTATCAG
AACCTGTCACTTTGTCAAATTTGTTTCCACTCATTTTATGTGCGGGTTCATGTGTGTGCGCATGTTTGTGTGATATGTCCATGCTGTGGTGTACAAGTGG
AAGACAGAGGGCAGCTTGAAGAGGTTGGTTTTCCCAACCATATGAGATCAAACAACTTATCAGGCTTTGTGAGCAGCAAGCTGGCCCGTTTCACCAGC
TCACACCTTCTTCTTTACCCTTTCCTTTTTATTGCCTTTATCTCTCCTACCTCCCCCTTTTTCTTTTTTGGGGGTGTGAGGAGAAGTAGTTGGAGACAGGGT
TTCTGTGTGTGTAGCCCTGGCTACCCTGGATCTCATTTGTACACCAAGCTGTCCTTGAACTCAGAGATCTGCCTACCTCTGTCTCCTAAATGCAGGGATTAA
AGGCATGTGTTACCACCGCCTGGCTCTTGAGATCCTTTTAAAGAAATACGTTATTTCAGCCCATTCTTTGGTTAGGCAATGGCTCCACATCTTTCAGATG
CCCTGCCCTCAAGGAGAGGCAAGATTCTAGGTCTGATTTGGGGCTTACTGATTGCTTGTTTTTGGTGGTGCTGGTAGTGGTCTTAATTTCTTCCGCTGAG
ATAGCAAAAAGAGATGTTTGACCCTGGAGAGAACAGAACCAGTCCCCAAATGCCACAGGTCTGGAGCAGCGGATCAACAAGGACTATCTAGGTTATA
AGACAAGGCATTCTCTCAGAGGTGGTCTTACTGGTCAGATACTGACAGTCTCAATCCATCCAGACAGTTATGGACATGGCGTGCAGTCCACTGGCTCTA
CCAGCACGGCAGTCTCTGGCAGTCCGTTTTTTTGTTTGTTTGTTTGTTTGTTTGTTTTTTTCTGCTCCTGCAGCCCCCATAGATGCATGAGCCAACTTCCTT
GTTCCTCTGCATCTTTGTGTCTGCCTCTATATCATTCTCATGGCCCAGAAGTTCCAAGGAAGATGGAACCAAGGCAGAGAGCTCAGTTTAACTTTCAGG
TAGATCACAGAGTTGGTGGGACGTGTCACGTATTTTCCTAACAACCTTCAGGATTGTGCAGAACCATCTCTGTTATTCTGGGAGGGCATCCATGGGCAC
AGCTCTTTGGTCCACCTCTGAGTCTCTTTGTATGCCCACATTCCCCTCTTGAATTGCCTGTGGGCATACCCCCATGCTCCCCACACCTGCCTGGGGACTC
CTCCCTATGTCATGTCCAGCAGGTCAACGTCTAAGATGCCACAAGCCCAAGGAGACTGTATAAACAGCAGTGCCCTCAGGTCCTCCCTGGGCATTGGTT
CTGTGTTGGACCCAGGCTGTGTACTTTAGGAAACTCTTCTTGGCCAGCTACAATGTCCTACACTCTGATCCCCCCCACCCCCACCAAGAGGGCCATGTG
TATCAGCTCAGGGGACACCTGGTATCTCCTGTTCCCTCTCCAGGTCCCTCCTCTGCCCCTCTCTTGTTCCCCAGCTGTTCCAGGTCAATGGCAGTGTCAC
ATACACTATGATTTGTCCCTCTGTCCAAAGATATACCCTGACGCTTCTGAACTTCTATTTAATCTTTCAGACACACACAAAGAGTCAATGCTTGGCCTCC
ACACAGTGATGGGCTTTATCCCAATGCATGCTGTGGGAGAGATTCAAGGCGCCGCTGTAGCCATGAGATTTATTCCTGGAGCAGTCAAGTACTGACTTA
CTGTCTCTGGTGCCTTAAACTGTCAGGATGCTAAGGTGGCTGGCTCAAAATATCCTCTCAGGTATCTGAGAGATGCTTCACCCATGTGGGGAGCCAAGA
CCACTGACTTTCAGGGCCTTCTCTGTGAGGGGTAAGGGAGATAATGGGGAGCCCTTCTCCCCCACTGACAGCTGGTTCTGTGTCAGTCCTTGTTGTACCT
CTCGGCTACTCCAGGAGGAAAGAAAAGTAAAAGCAAGAGTTAGCGGAATTCAAGAGATTCCTTCTCCTTTTCCTCCATCTTGAACAGAATGTTTCAGGT
TGAGCCCAGCTGGTAAAAAGTCTCCCTCCTCCCTTTTACTCATTTCATTCCCTTTGGGGTGCTCTTGGGTGAAGACATGATATCTGGAGCTACAGTAGCC
CAGCTGCCTTAAGACCACGAGGACAGGCGCTGTGATCGCAGTGAAAGCCACGGACGAAGGATGCACAAGTGCCTGCCTTAGTGCTCCTTACTCTCTAG
CTCAGTGGTTCTCAGCCTCCCTAATGCCGCAGCCCTTTAACACAGTTCCTCAGGTCGCGGTGACCCTGAACCATAAAAATTATTTTCTTGTTACTTCCCA
ACTGTGACTTTGTTACTGTTCTGGATCCTAATGTGAATATCAGATATGCAGGATATTTGAGATGTGATTCCCCCAAAGGCTTTGTGACCCACAGATTGG
GAACTGCTGCTCTCTACTCTAGCTTTGCAGTAAAAGCACCTATGGTTGGAGTCATTGCAAGATCTCTGCAGCTCCCTGTCCAGCAAGGGTAACCCCAGG
CTCCTGAGAGATCCTGCCTCAAAACAAACAAACAAATAATTGAAATGGGTAGAGCACTTGCTGAGCAAGCCAGAAGACTAGGAGAGCAGAGTCCAGA
TCTATTCACCCATGCGAGTCCAGGGCAGGCATGGTAACCAGACTGTAATCCCAGAACACAAAAAGTAGAGACAGGATCCCAAAGCAAGCTGGCTAGA
TAAACGAGCTCAACTGGTAAGCTCTAGGTTCAAGTAAAAGGCCTTAACTCAGTGTATATGGTGGAGAGCAGCTGAGGGACACATCTGGCCTCCACACA
TCGGTACCCATGCACACCCACACACATGCAAACATGTATACACACAGATGCGTACCGCATAAGCAAAATTTTTTAAAAAATTAAGTATATGGTGCCTG
AGGATCAACACCATAGCTTGTCCTCTGAGCTCCACATACATCTTTACACATGGGCACACTTGGACATCTAGTACCCGTGCACACACACACAAAGATGG
ATATCAAAGTCATGCTTTCTACCTGTCAGATTGTCCTATCTGCTACTAATAATCCTCATACCAGAAAAATAATAAAATAGAGTGTTCTTTAGTTTGCTTG
TTTTTTGTTTTTGTTTTTGTTTGCTTTGGTTTTATTGTTATTTATTTATTTATTTATTTATTTATTTATTTATTTATTTTTGATATGGTCTCGAATATGTACCCTTGGC
AGGCCTGGAACTCACTATATTGATCCAGCTGAACTTGAACTCACAGAAATCTGCCTCTGTCTCTCAACTGTTGTTATTAAAGGCATATACTACCACACCT
AGCTTGAAATAGACCTCTCATTTTCATTATAACAAGATAGGCTCTATATAATTATATTGTAAGCTTTTTGACCAATGAATGTGTAAACTCTAAAGGTTAT
TCTTATGGTTTGGATGTGACATGTCCTGTCCCACACTCCCTTTTACCCAGGCTCGTGTGTTTTGAACATTTAGCCCCTCAGCTAGCAGATCTATTTTGGGA
GGCCGTGGAACCACTGGCAAATAGGACCTTGTTGGCTGATATAGATCACAGAGGCACACCCTGGGAGGTCACAGCCCACTTTATTTCCTGTCCTACCTC
TGCTTCCTGGTCACTCTTTGTGAGAGGCTGCTGTTATGATAGCCACCATCGCAGACCCCACCTGCTCTCCAGCCACCATGCCTTGGTGAATGGTATTCAC
CTCGCATCCACGGGGCCTCATGGTCTACCATGGTGAATTATAGTCCCTGAGCTGTGAGCCAGAGTAAATCCTTCTTCCCTGAAGTTACTCCTGTCGGGT
ATCGTCACAGAGAATAGAAAAGTAATGACCACAGTCATATATCTAATTACAGGAATCTGGGAAATGACACATAAATCCACCCAACCAACACACACGG
AAAGCCAGCACAAAGCCAGTGCTGGACACAGGGGCCAGTGAGGTGCAGTTTTCTTGGTTACTAGTTAATTAACTGGGGAAACTTGGCAAAGGCACTCA
GCGTCATAAAGCAAGGCAGAACCAGAGAGATGCCAAGAGGGGTCCACAAGAGGGCTGGGCCAGCACACAGGCCAGACTTCATTCCAGCTTGTCTGGA

AAGGCTGACTAGCATCCAGCTCTGGTTTCTCAGTTGACAAGAAAGGGGGAGCTAGAACAATGAAACCTAAATACCCCGGACTCCACCCTCCCCTCCTC
CTGGCTATGCACCTCCTTCTGCCTCAGTCAGCGCTCCCTTCACTCTGGAATCCATGTAAAAAAATTTATTTCTCTCAGTAAAATAAAATCACAGAATTCT
CCCTTCAAAATTTCCTGTCTCTGATCTATCCAAAAAACTCTAGTGCCAGGAAGGAATGGGGTCGCTTTCATGGGAGAGACGTTTGTTATGTAACGCTCC
AATGCCGGGAGAGCCAGTGGACGCGCCGCTGGCGGGTTCTGTCCAAACTTTGCAAGAGGCAAACATGTTTCGCTCAAAAGGACCACAGAACCCTCCTA
AAACGTGAACGAGGAGCAGGCTTACACCACAGGAAAAGGGATTTTGTGGGTTTGGTTTTTAAGAAAGAACAGACTTAGTCAAGAAAGGCTTAAGAGC
TGCCCAACCAACCCTAGATCCTGGTAAATAAATAGCCTGAGATTTACATCTGGAAGGTGGAAGTCTCAAAGCCAGTCTGAATTATATCAAGGAACTGC
AAACACATTTCAGAAGTATAAATACAGTCATCTCATTTGCTGATACCATTACAGAGACAGGTAAATAGAAGCGATTACATCCCAGTTTCTGGAAGGAA
TCATTTAGTTATTTACAAATTAAGTTTTAAGTGGCCGGCCTACAACAGCTGCGGAGAAGATAAACTGTTAGAAAGCTATATACTTTTTAAAAAAGCTCT
TGAGTACAGCTGTATTACCTGTAGAACGCTGGTTGGTGTAGCCCTGTGGGAGAGACAGCTTAATTTTCTGCTGGGAAATGACATGTGAGAAACGCACA
TGGTTTCTGGCTTGGAACCTGGAAAGAACAGATACACACATCAGTTTCAAAGGGGCCTTGGAAATCATTCACACTTCAACGGCAGTGTGCTGGGTAGT
GTCTCTCAGTTTAATAGTTACTTAGTATGGGCTGGGGAGGTGGCTCATTGGGTACAGTAAGCATGGGGACCTGGGTTCTGACCCCCCAGGACCTAGGTA
AAAAGCTGAGGACAAGCGTGCAGCTCTGTCATCCCATGCTACAGAGGCCACAGAATGCATTCTGGCCAGTCTAGATGAATGACTGAGCTCCAGGTTCC
TGAGAGACCCTGCCTCAAAAAGTAAAGTGAGGAGTGATTGAGGAAGACATTAGATAAACTGTGGTCTCTATCTGCACACATGCATCTGCATACATACA
TCTACTACACCACACACACACACACACACACACACACACACACACACACACGCACACACGCACACACGCACACACGCACACACACAAACACACATGCAC
ACTCAGAGAGAGAGAGAAGACAGACAGACAGACAGACAGATAGATAGACAGACAGACAGAGACAGAGAGCAAGGGAGAGAGAGCAAATTGAAGAG
AGAGGAGAAAAGAAAATCTATAGCAGAGCTTGGGGATTTACCTCAGTGGTAGAGGGCTTGTGTGGCAAGCATGGGGCTTAAGTTCCATCCCCAGACCT
GGGGCTAAAAATTTTATTTGAAATGCACAAGATGATGAGATTTCTCCCCTCCCCCCATTTAACATTCCTTGCTTCCTCCCCCCTTTTTTTCCCCACAATGG
TCCCTGACTATTGTGTAAGTGTGGCCACGGGCTGGGCAGACAGAGAAAGGATTCTTACTGCAGGCCTGCATTCAGCGAAGTGGCATGGAGAGTGTAAT
TGGGCCATGGGTAATTCTTTAAGCTCAAAGTCGAGATCAGTTTCATTTGCATATCTATCTTGAGGAATTATGGGATACCCAGAGCTATTTCTAGAATGCT
ATTTGCATAATTGTTCTAATCTTTTATGAATACGGCGACATGTGTGGTCTGACAAAGGATTTTTGAAAGAAAATTTGTTAAGTTGCTTTATGAGTTTGTA
AAGATATTAGAATTAAATAGTGCTAGGTATATATTTAATAACCTGTTTCTATTGAAGGAACACAATTACATAAAGCCATTAGTCTGCTGCCAATGGCTT
CTTACCTTTGCCATTGTTGTTCCCCGAAAGTTTATCATTTAAAGTTGATTTCTCCTTCACACCCCACCACCAACACTTCTTAGAAAGAATTACATTCACTT
AAACACCATGAGAAAATGTACAGTTGTTTTGTGACTCTCTGATTCCCTGCTCCCTGAATTTCTTAATCTGTGGGCATTCGACATGTTTCCAGCTTTGGAC
ACTAGCTACAAGACAGACAGGCTCACAGTCTTTGTGGTCTTTTCTATCCTGGGGTGCTCATGATCAAACTACTTGAGTTTCACCATTTCTAAAAAGATCC
TTGCTCCACTCAGCAATTTCTTCTTCTTGTGGGACCATCAGGACTACGAGGAGGAAATGCATGCAAGTCTAGTCCTCCATAGTTTGAAAATTCTCAGAC
TTCAGGAGGGAGAACTCCTGTCTGACCTGTGACCACTGTAACTCTCCAGCCATTTCCTCTCCTCCATTCTGGTTCCATTCCTGAGCTGGCCAACCCCAAA
AAGACTGTTGAGGTATGGTCTGTTGCTATGTATTGGAAAGCTAAATTCTGTGCTCAAAGCAAATTCTCACATTTATAAGCTTTTGTTGTTCCTTAACTTA
AAACTATTGGTTAAATAAAATTGGCTACACCCGATTATTGAGCAATTAGAATCTGGTAGGTCTTGAGTTACCTGGTTGGGGGTAGAGAAGCTGGAAAG
GAGAGGAGAGACCACGAGGAGAGAGGGGAGAAGACACCACTAGAGGAGATGAACCATGAGCACATAGCCAGGAGAGACAGCAAGTATTTGAGAAC
ACTGCTGGGGAGGTAGCCAGGCCAGCAGTTAGAAAAGCAGATTAGGGGTTACCCCTTAATTGCCAAAGCCAAATAAAATAACCAGAGTCTGAGTCTCA
TTTATTTGTAAGCTTACATTGGTTCAGCCCCACTTTTACCTCTAGTTCACATTAAAGGCTAAGACCTCCTGTCCTTGTTGTTGCCAACTGTGTGCCTGTTT
GGCTATCACCATGTACCCACATAAAATCTAGGTACACCCCTTTGGCCTGTCACCAGTCTCAACACCAAAACCAATCTGAGGTGTGGGGTTGATCCCACT
TTCTGAAGGACAATCACTACAAAATGAGCATCTTTATTTTTAAATTTTGGTTTTGAGACCAGGTAGAGCCTGGTCTGCAAGTGCTACTTAAGTAGCCTC
AAACTCAGTATGTAACAGGAAGACCTTGAACTCCTGATTCTCCAGCCTCGATCTCCTGAGTACTGGGATTACAGCTATGTGCCACTATACCTGGCCACT
AGTGTTTTATTTATTTATTTGTAATGATTTATTTATCTTTATTTTATGTGTATTGTTTCTTTGTCTGCAGGCATGTCTGTGTACCATGTGTAGGTCTGGTAA
CCATGGAAGCCAAGGTCCTCTGGAAGAGTGCTGCCATGGTTTGTATATGCTTGGCCCAGGGAGTGGCATTATTAGGAGGTGTGGCCTTGTTGGATTAGG
TGTGTCACCATGGGCATGGGCTTAACACCCTCACCCTAGCTGCCTGGAAATCAATATTCCACTAGCAGCCTTCAGATGAAGATGTAGAACTCTCAGCTC
CTCCTGTACTATACGTGCCTCAATGCTGCCTATCTCCTGCCTTGTAATAATGGACTGAACCTCTGAACCTGTAAGCCAGCCCCAATTAAATGTTGTTCTT
TATAAGAATTGCCCTGGTCATGGTGCCTGTTCACAGCAGTAAAATCCTCACTAAGACAAGTGCTCTTAACCACTGAGCCATATCCCTAGCACCCTATAG
TGATTTTAAAAGGTAACACACTGAAATTTAAAGTGTAACAAAATGATAACCCATTAGAAATACTCATTAGATTAGGAAAGAGATGATGGAGATGTCTA
CCAAGATGACTCCAAACAAGGGCCACTTGAATGCTTCTTCCTGCATTGAGGGTGTGTGTTTCAGGAGGCCCCAGGGATTCTGGCTGTTAAGTCAACTCA
ACCAGCTTCCCCAGGTCCTTCACTGCCTGGGCCTTATGCCAAATGTACTCATCTCTGTTGGAGAGGAAGCTCAATTTTCTTTTAGTAAAGTAAGGGAGA
TCACATCTGCTCCCTTCCGCTGCTCTGAATTTAAGTCACACAGTCACACGTGAACAATCAGTAAACGGTCTCTGAATGAGCTGTAGAGGACAGCTGCGA
TCCATAGATCTCTTCTACTAGCCATAGGTTTCTCAGCTCTTAATGAAATGAAAGAAGAAAAGTGATAGGGAATATTAACATTGGCCATGCAAAGGACT
AATAATTTATTTCTGTATCCTCATAAAAACAAAGCAAATACTACTTGCTATGCATCTCGTGTTCGGGGAGCACCTTTTGGCTGCATTGCACCTTGGTAGA
TGGCTCGATGATGAGAGGGGATAATCTCAGGGTAACACAGGAAGCTGGAGTGGAGTCATTAGGAGGACCCAAGTTTGCTCTTTAATAAAAACCCCTTA
GTTAGGGTTCCTATGGCTGTGACGAAACACCACGAACAAGGCAAGTTTGGAAGGAGGAGTTTATTCACATTACTGTTCACTATTAAAGGAAGTCAAAG
CAGGAACTCAAACAGGGCTGGAACCTGGAGGCAGGAGCAGATGCAGAGGCCATGGAGGAGTGCTGCTTACTGGGTTGCTCATCACGGCTTACTCTACC
TTACTTCTTTCCTTCCTTCCTTCCTTCCTT
